# Topology and Dynamics of the Zebrafish Segmentation Clock Core Circuit

**DOI:** 10.1371/journal.pbio.1001364

**Published:** 2012-07-24

**Authors:** Christian Schröter, Saúl Ares, Luis G. Morelli, Alina Isakova, Korneel Hens, Daniele Soroldoni, Martin Gajewski, Frank Jülicher, Sebastian J. Maerkl, Bart Deplancke, Andrew C. Oates

**Affiliations:** 1 Max Planck Institute of Molecular Cell Biology and Genetics, Dresden, Germany; 2 Max Planck Institute for the Physics of Complex Systems, Dresden, Germany; 3 CONICET, Departamento de Física, Universidad de Buenos Aires, Buenos Aires, Argentina; 4 École Polytechnique Fédérale de Lausanne, Institute of Bioengineering, School of Life Sciences, Lausanne, Switzerland; 5 Institute for Genetics, University of Cologne, Cologne, Germany; 6 École Polytechnique Fédérale de Lausanne, Institute of Bioengineering, School of Engineering, Lausanne, Switzerland; University of Dundee, United Kingdom

## Abstract

During vertebrate embryogenesis, the rhythmic and sequential segmentation of the body axis is regulated by an oscillating genetic network termed the segmentation clock. We describe a new dynamic model for the core pace-making circuit of the zebrafish segmentation clock based on a systematic biochemical investigation of the network's topology and precise measurements of somitogenesis dynamics in novel genetic mutants. We show that the core pace-making circuit consists of two distinct negative feedback loops, one with Her1 homodimers and the other with Her7:Hes6 heterodimers, operating in parallel. To explain the observed single and double mutant phenotypes of *her1*, *her7*, and *hes6* mutant embryos in our dynamic model, we postulate that the availability and effective stability of the dimers with DNA binding activity is controlled in a “dimer cloud” that contains all possible dimeric combinations between the three factors. This feature of our model predicts that Hes6 protein levels should oscillate despite constant *hes6* mRNA production, which we confirm experimentally using novel Hes6 antibodies. The control of the circuit's dynamics by a population of dimers with and without DNA binding activity is a new principle for the segmentation clock and may be relevant to other biological clocks and transcriptional regulatory networks.

## Abstract 2

By combining biochemical, embryological, and mathematical approaches, this work uncovers an important role for protein-protein interactions in determining the dynamics of the somite-forming segmentation clock in vertebrates.

## Author Summary

The segmented pattern of the vertebral column, one of the defining features of the vertebrate body, is established during embryogenesis. The embryo's segments, called somites, form sequentially and rhythmically from head to tail. The periodicity of somite formation is regulated by the segmentation clock, a genetic oscillator that ticks in the posterior-most embryonic tissue: for each tick of the clock, one new bilateral pair of segments is made. The period of the clock appears to determine the number and the length of segments, but what controls this periodicity? In this article, we have investigated the interactions of three transcription factors that form the core of the clock's regulatory circuit, and have measured how the period of segmentation changes when these factors are mutated alone or in combination. We find that these three factors contribute to a “dimer cloud” that contains all possible dimeric combinations; however, only two dimers in this cloud can bind DNA, which allows them to directly regulate the oscillatory gene expression that underpins the periodicity of segment formation. Nevertheless, a mathematical model of the clock's dynamics based on our experimental findings indicates that the non-DNA-binding dimers also influence the stability, and hence the function, of the two DNA-binding dimers controlling the segmentation clock's period. Such involvement of non-DNA-binding dimers is a novel regulatory principle for the segmentation clock, which might also be a general mechanism that operates in other biological clocks.

## Introduction

Rhythmic phenomena are widespread in biology and the control of their timing is fundamental to many processes. Yet how the dynamics of genetic circuits that control rhythmicity are regulated remains poorly understood. The segmentation clock is an attractive model system to address this question. This gene regulatory network operates in the presomitic mesoderm (PSM) of developing vertebrate embryos and generates transcriptional oscillations that direct the rhythmic and sequential formation of body segments in concert with embryonic elongation [1]–[3]. Many components of the segmentation clock have been identified in the last decade [4],[5], but how they interact to produce oscillations remains unclear. The oscillations of the segmentation clock are most easily observed at the tissue level [6], but they arise on the level of single cells [7].

Current models for the origin of single cell oscillations in the zebrafish segmentation clock posit a negative feedback loop involving the *her1* and *her7* genes [8]–[10], which encode members of the Hairy and enhancer of split related (Hes/Her) family of basic helix loop helix (bHLH) transcriptional repressor proteins. Specifically, it has been proposed that oscillations arise through the auto-repression of these genes via a mix of Her1 and Her7 homo- and heterodimers, all of which have identical properties. This model is consistent with the reported redundant functions of *her1* and *her7* in somitogenesis [11],[12] and the observation that overexpression of either *her1* or *her7* leads to repression of both genes [13]. Furthermore, direct binding of Her1 homodimers to sites in the *her1* promoter has recently been shown in vitro [14]. However, biochemical evidence for the other regulatory interactions proposed in this model is still lacking. It is also not clear how the proposed promiscuous protein-protein interactions and the equivalent functions of the resulting dimers in the current model can be reconciled with the reported distinct loss-of-function phenotypes of either gene. Knockdown of *her1* results in segmentation defects preferentially located in the anterior trunk, while *her7* knockdown in contrast leads to fully penetrant posterior segmentation defects [11],[12].

The period of the oscillations of the segmentation clock, in concert with embryonic elongation, determines the number of embryonic and adult segments, and is therefore of key importance in determining a species-specific body plan [15]–[17]. How the period of single-cell oscillations is controlled molecularly is not known. The cyclically expressed genes *her1* and *her7* have been proposed to differentially regulate the period as a consequence of different protein production delays [10], but this has not been tested experimentally. The only bHLH factor gene for which there is experimental evidence for a role in controlling the period of oscillations is *hes6* [17]. *hes6*, in contrast to *her1* and *her7*, is not cyclically expressed, but displays an FGF-dependent posterior-to-anterior expression gradient in the PSM [18]. In addition to its role in setting the period of the segmentation clock, *hes6* contributes to stabilizing the transcriptional oscillations of *her7* and *her1* in the PSM [17],[18]. The Hes6 protein physically interacts with Her1 [18], but the interactions of Hes6 with other cyclic clock components have not been explored, and consequently, the molecular mechanism by which Hes6 controls the period of the oscillations is not understood.

Here we map the topology of the regulatory interactions between Her1, Her7, and Hes6 and DNA sequences in the promoters of cyclically expressed genes. We find that all of the possible dimers between Her1, Her7 and Hes6 form, but only Her1 homodimers and Her7:Hes6 heterodimers have strong DNA binding activity and target similar DNA sites. Using our experimentally determined network topology, we develop a simple mathematical model that can account for single and double mutant phenotypes that we observe. In this model, sequestration of monomers into dimers without DNA binding activity underlies the observed distinct phenotypes of genetic mutants. A surprising prediction of this model is that Hes6 protein levels oscillate post-transcriptionally, and we confirm this with a novel Hes6 antibody. Together, our results lead to a major revision of the current model of the core circuitry of the zebrafish segmentation clock and emphasize the importance of the properties of the Hes/Her protein-protein interaction network in controlling the clock's dynamics.

## Results

### Hes/Her Dimers Form Promiscuously, But Only Her1 Homodimers and Her7:Hes6 Heterodimers Have DNA Binding Activity

**Figure 1:**
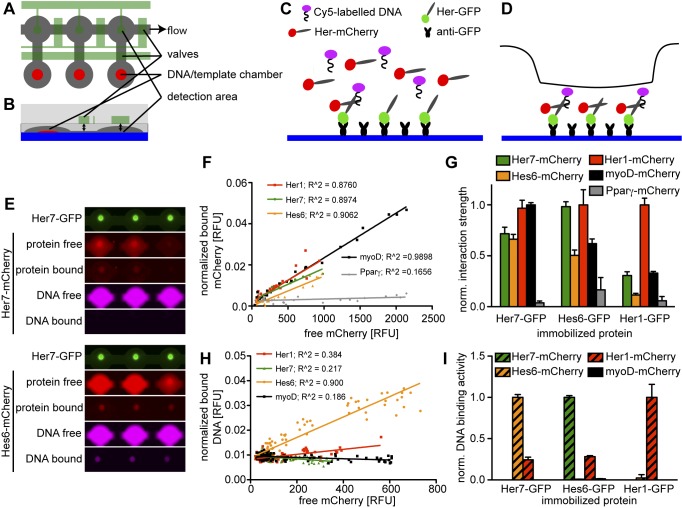
bHLH proteins in the segmentation clock interact promiscuously, but only few dimers bind DNA. (A–D) Schematic of MITOMI setup. (A) Top view of three unit cells, (B) cross-section of one unit cell. Flow layer in grey, control layer in green, supporting glass slide in blue, and spots of expression template or target DNA in red. (C) GFP-tagged Her protein is immobilized on the slide surface with anti-GFP antibodies. mCherry-tagged Her protein is expressed in the chamber in the presence of Cy5-labelled DNA and allowed to bind to the immobilized protein. (D) The valve over the detection area (black line) is actuated to displace unbound protein and DNA and detect specific interactions. (E) Representative images of six chambers of a microfluidic chip with Her7-GFP (green) coupled to the chip surface with Her7-mCherry (upper panels) or Hes6-mCherry (lower panels) expressed from expression templates spotted on the chip in the presence of a Cy5-labeled DNA oligomer. Both Her7-mCherry and Hes6-mCherry interact with Her7-GFP, but only the combination of Her7-GFP and Hes6-mCherry binds DNA. (F) Quantitative analysis of protein-protein interaction of Her7-GFP with Her1-mCherry, Hes6-mCherry, Her7-mCherry, myoD-mCherry, and PPARγ-mCherry. mCherry signal in solution is plotted against the bound signal in the detection area. The slopes of the linear fit to the data points for each mCherry-tagged protein provide relative measures of their interaction strengths with Her7-GFP. (G) Relative strength of protein-protein interactions between Her1, Her7, Hes6, myoD, and PPARγ assayed as in (F), with Her7-GFP (left), Hes6-GFP (middle), or Her1-GFP (right) coupled to the chip surface. Bars represent the slopes ± standard error of the linear fits as shown in (F), with the slope of the strongest interaction normalized to one for each experiment. (H) Quantitative analysis of DNA binding activity of Her7 homo- and heterodimers. mCherry signal in solution is plotted against signal of bound DNA in the detection area; the slopes of linear fits to the data give a relative measure of the DNA binding activity of the different bHLH dimers. (I) Relative DNA binding activity of dimers formed between Her1, Her7, Hes6, and myoD assayed as in (H), with Her7-GFP (left), Hes6-GFP (middle), or Her1-GFP (right) coupled to the chip surface. Bars represent the slopes ± standard error of the linear fits as shown in (H), with the slope of the strongest interaction normalized to one for each chip.

To investigate the topology of the regulatory network formed by Her1, Her7, and Hes6 we first asked which dimers form between the three factors. Co-immunoprecipitation suggested that all possible dimers form rather promiscuously, and even the bHLH containing factor MyoD, but not the negative control non-bHLH protein PPARγ, was co-purified by Her1, Her7, and Hes6 to a similar extent (Figure S1). To verify these findings in an independent setup and to investigate the DNA binding activities of the different dimers, we employed a microfluidic platform and mechanically induced trapping of molecular interactions (MITOMI, schematically depicted in Figure 1A–D) [19]. In this system, GFP-tagged Her proteins are immobilized on the surface of a microfluidic chip, and the pulldown of mCherry-tagged proteins is used to assess protein-protein interactions. The DNA binding activity of the resulting dimers can be investigated in the same assay by adding a DNA fragment that is labeled with a different fluorophore. We therefore coupled GFP-tagged versions of each of our three Hes/Her proteins to the chip surface, expressed different mCherry-tagged bHLH proteins in individual chambers of the chip [20],[21], and added to the expression mix a Cy5-labeled DNA fragment that contains the 12 mer CGACACGTGCTC from the *her1* promoter. We chose this sequence because it has previously been shown to interact with Her1 in electrophoretic mobility shift assays [14]. Varying the amount of expression template spotted in the reaction chambers allowed us to titrate the concentration of mCherry-tagged protein in each chamber (Figure 1E,F). Protein concentrations reached by on-chip expression are below the dissociation constant K_d_ reported for the prototypical bHLH proteins E12 and MyoD [22]. At these concentrations, the amount of bound protein for any concentration of free protein is approximately inversely proportional to the dissociation constant [23], and therefore the slope of the linear fit to the plots of the free mCherry signal against the normalized signal from bound mCherry (Figure 1F) can be used as a measure for the relative affinities of protein-protein interactions.

When we coupled GFP-tagged Her7 or Hes6 to the chip surface, the four mCherry-tagged bHLH proteins showed a 4- to 20-fold higher relative affinity to the immobilized protein compared to the negative control PPARγ. The differences in relative binding affinity between bHLH proteins, however, were at most 2-fold (Figure 1F,G), in agreement with our immunoprecipitation experiments. In this assay, GFP-tagged Her1 coupled to the chip formed mainly homodimers. Heterodimerization of Her1 with Her7 and MyoD occurred with approximately 3-fold lower relative affinity, but was still significantly stronger than binding of PPARγ to Her1 (*p*<0.01), and only the strength of Hes6-mCherry with Her1-GFP was not significantly different from that of the PPARγ–Her interaction in this assay (Figure 1G). Because this latter finding is in contrast to the results of our co-immunoprecipitation experiments, and the interactions of Hes6-GFP with Her1-mCherry in MITOMI experiments, we suspect that steric factors hinder the formation of Hes6:Her1 heterodimers when Her1 is coupled to the chip surface. Taken together, our protein-protein interaction studies indicate that interactions are non-selective between the Her1, Her7, Hes6, and MyoD bHLH proteins.

As a next step we analyzed the DNA binding activity of all homo- and heterodimers detected in our protein-protein interaction experiments. In the presence of mCherry-tagged Her7 or MyoD, GFP-tagged Her7 immobilized on the chip surface did not bind the DNA sequence derived from the *her1* promoter, whereas the presence of Her1-mCherry conferred weak binding, and the presence of Hes6-mCherry conferred strong DNA binding activity (Figure 1E,H,I). A similar situation was observed for Hes6-GFP, which bound the same DNA fragment strongly in the presence of Her7-mCherry, weakly in the presence of Her1-mCherry, but did not bind DNA in the presence of Hes6-mCherry or MyoD-mCherry (Figure 1I). Her1-GFP coupled to the chip surface bound DNA in the absence of any coexpression partner, presumably as a homodimer (unpublished data), and this binding was further increased by the presence of Her1-mCherry, but not Her7-mCherry or Hes6-mCherry (Figure 1G). These findings suggest that, while protein-protein interactions between Hes/Her factors are promiscuous, the DNA binding activity of the resulting dimers is restricted: Of the dimers formed between the three transcription factors investigated here, Her1 homodimers and Her7:Hes6 heterodimers bind most strongly to the target sequence from the *her1* promoter.

### Her1 Homodimers and Her7:Hes6 Heterodimers Bind with Equal Preference to Multiple Sites throughout Cyclic Gene Promoters

**Figure 2:**
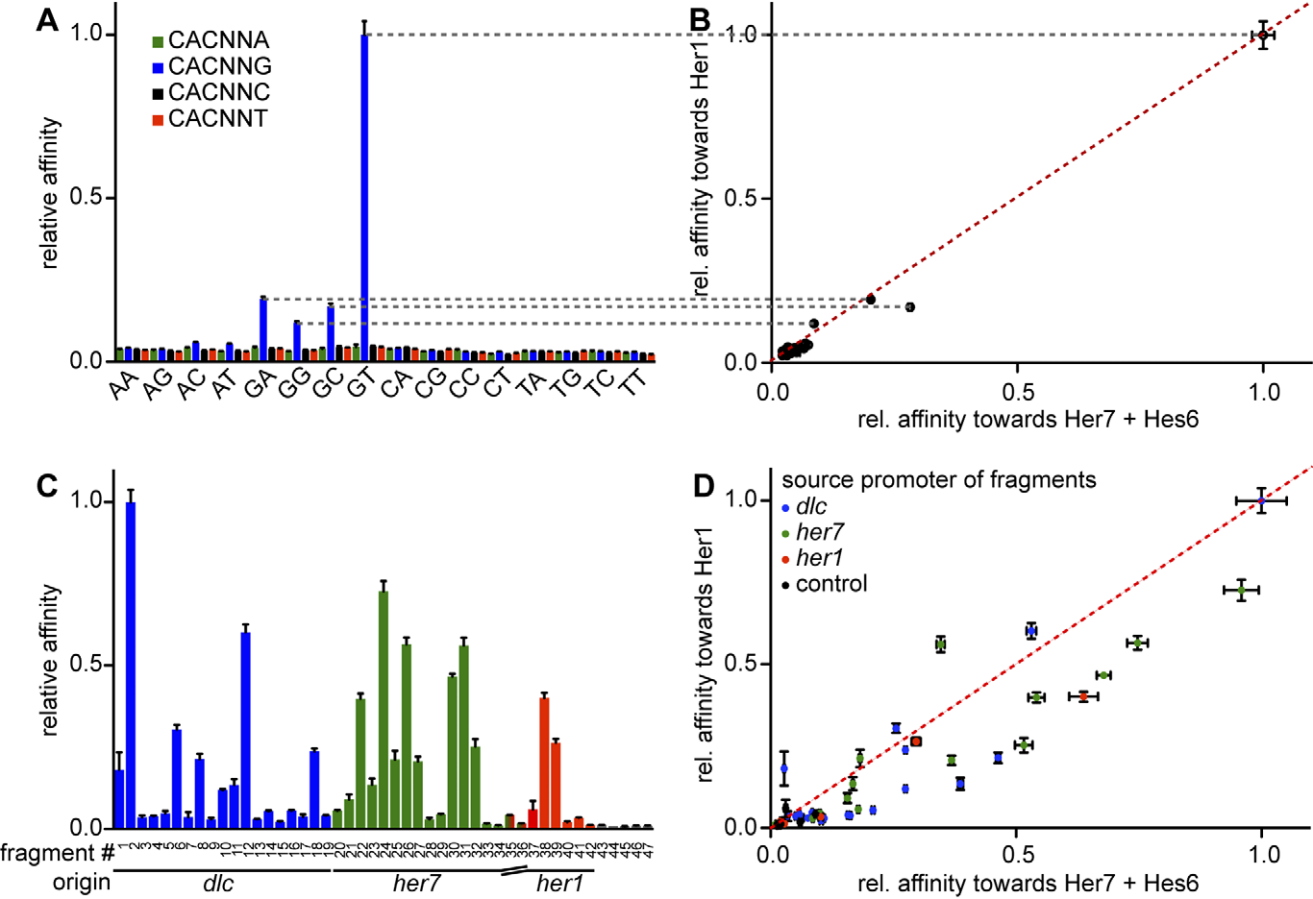
Her1 and Her7:Hes6 bind similar DNA target sequences. (A) Relative binding affinities of the 64 permutations of the sequence CACNNN to Her1. Concentration series of labeled oligonucleotides were deposited on microfluidic chips and their binding to Her1-GFP immobilized on the surface of the chip measured by MITOMI. Bars indicate the slope of the linear part of the free-versus-bound-DNA plot for each sequence with the slope of the highest affinity site normalized to one. Error bars indicate standard error of the fit. The first two variable bases of each permutation are indicated on the category axis, and the last base is color coded. (B) Relative affinities of sequences in the CACNNN library to Her7:Hes6 heterodimers were determined as in (A) with GST-tagged Her7 coupled to the chip and untagged Hes6 in solution, and plotted against their relative affinities to the Her1 homodimer. (C) Relative affinities of all NNNCACGNGNNN sequences present in the *dlc*, *her7*, and *her1* promoters to Her1 were determined as in (A). Nucleotide sequences corresponding to fragment numbers are listed in Table S1. Color code of bars indicates the promoters from which each sequence is taken: blue, *dlc*; green, *her7*; red, *her1*; black, control sequences lacking an H-box. (D) Relative affinities of the Her7:Hes6 heterodimer to each sequence of the NNNCACGNGNNN library were determined as in (C) with Her7 coupled to the chip and Hes6-mCherry in solution, and plotted against their relative affinities to the Her1 homodimer.

To identify additional potential Hes/Her binding sites in cyclic gene promoters and to test whether Her1 homodimers and Her7:Hes6 heterodimers have similar or distinct DNA binding preferences, we sought to systematically investigate the DNA binding specificity of the two dimer species. We did this again using MITOMI, but now deposited increasing concentrations of different labeled DNA sequences in the chambers of the microfluidic chip. For low DNA concentrations, the amount of bound DNA for any concentration of free DNA is inversely proportional to the dissociation constant K_d_ of the complex [23]. The slopes of the linear fits to the data points for individual sites can therefore be used to compare relative binding affinities of a set of sequences to a given protein. To identify the consensus binding sequence of zebrafish Hes/Her factors, we first tested binding of all 64 permutations of the sequence CACNNN by Her1 homodimers and Her7:Hes6 heterodimers. We found that both dimers prefer the consensus site CACGNG (with N = T conferring stronger binding than A, G, and C) over all other sequences (Figure 2A,B). We term this common consensus binding site of Hes/Her proteins the H-box.

Although both Her1 homodimers and Her7:Hes6 heterodimers prefer binding to the H-box consensus site, it is possible that the two dimers prefer distinct bases flanking the core hexamer and thereby preferentially regulate different genes in the segmentation clock. To test this idea, we created a library comprising all H-box sequences flanked by three nucleotides 3′ and 5′ as they occur in the genomic context within 20 Kb of the *her1/her7* locus and 12 Kb of the *dlc* cyclic gene locus (see Table S1 for sequences and localization of these H-boxes). We first measured relative binding affinities of the sequences in this library towards Her1 homodimers. We detected a characteristic profile of sites with a range of affinities (Figure 2C, Table S1). In this library, sequences that contain the core hexamer CACGGG or CACGAG were bound at most 5-fold stronger than control sequences that lacked an H-box consensus, whereas all H-box sequences with a more than 5-fold stronger relative affinity to Her1 homodimers compared to control sequences contain either a CACGTG or a CACGCG core hexamer (Table S1). This suggests that the relevant H-box in vivo is CACG[T/C]G. Furthermore, we found that the bases flanking the core hexamer influence binding affinity. Certain flanking bases reduced the affinity of sites containing the optimal CACGTG consensus by more than an order of magnitude (compare sequences 2 (TGGCACGTGTCC) and 7 (CATCACGTGAAA) from the *dlc* promoter, Table S1). In the appropriate sequence contexts, the CACGCG hexamer was bound almost as strongly as the highest affinity CACGTG-sites (e.g., sequence 26 (GGGCGCGTGCCG) from the *her7* promoter, Table S1). With a few exceptions, H-boxes flanked by G or C were generally bound more strongly than those flanked by A or T. Importantly, we found that several H-box sites from the *dlc*, *her1*, and *her7* promoters were bound by Her1 homodimers with comparably high affinity (Figure 2C), suggesting that Her1 can potentially regulate all three genes.

To test whether this was also the case for Her7:Hes6 heterodimers, we determined the relative binding affinities of the same sequences to Her7:Hes6 heterodimers and plotted their values against those determined for binding to Her1 homodimers (Figure 2D). If the target site specificity of Her7:Hes6 heterodimers was distinct from that of Her1 homodimers, and Her7:Hes6 heterodimers preferentially regulated one of the three cyclic genes, we would expect that the datapoints corresponding to sequences derived from different promoters cluster together. However, this was not the case: the datapoints were evenly distributed along the diagonal of the plot, irrespective of their origin.

Finally, motivated by our finding that Hes6:Her1 and Her7:Her1 heterodimers weakly bound an H-box sequence from the *her1*-promoter (Figure 1), we wanted to test whether these heterodimers target a distinct subset of H-boxes. We therefore co-expressed Hes6 or Her7 with Her1 coupled to the chip. This did not change the affinity profile of Her1 (Figure S2), which suggests that Her1:Hes6 or Her1:Her7 heterodimers do not bind to a subset of H-boxes distinct from the ones bound by Her1 homodimers. Combined, these results indicate that the strongest DNA binding is from Her1 homodimers and Her7:Hes6 heterodimers; these have similar DNA binding specificity, and each dimer has the potential to directly repress *dlc*, *her1*, and *her7*.

### Her1 Homodimers and Her7:Hes6 Heterodimers Target the Same Cyclic Gene Promoter Fragments in the Yeast One-Hybrid Assay

**Figure 3:**
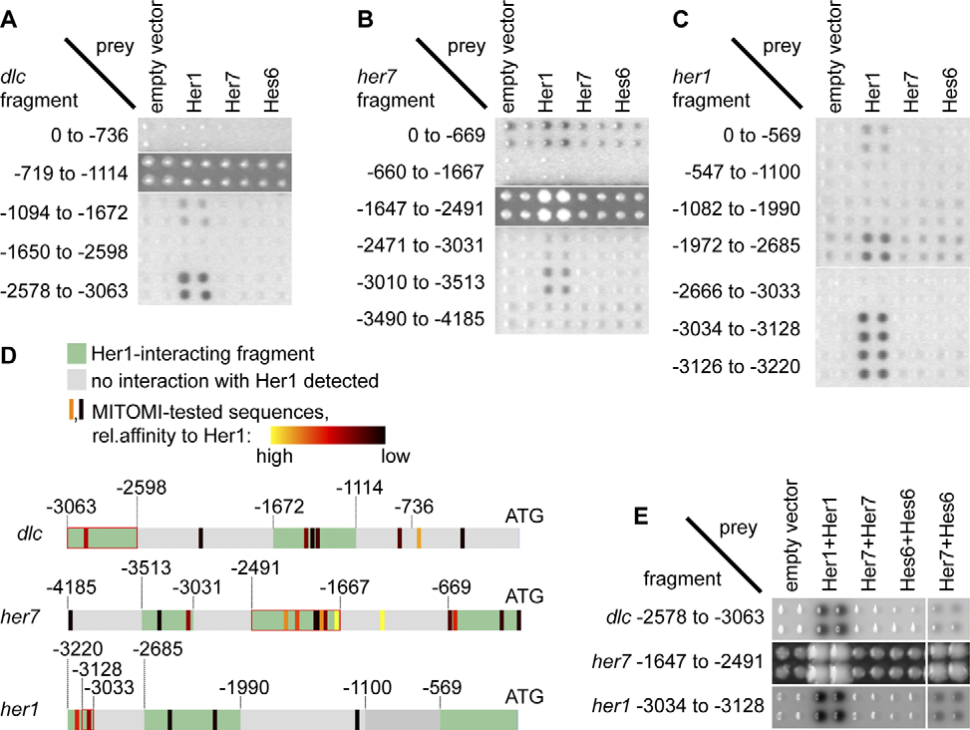
Interaction of Her1 and Her7:Hes6 with *her1*, *her7*, and *dlc* promoter fragments in a yeast one-hybrid assay. (A, B, C) 18 yeast strains carrying promoter fragments of the *dlc* (A), *her7* (B), or *her1* (C) genes coupled to His- and lacZ-reporter genes were transformed with plasmids encoding N-terminally Gal4-AD tagged Her1, Her7, or Hes6. Protein-DNA interactions were detected by staining for β-galactosidase, except for two fragments from the *her7* promoter (−1647 to −2491) and *dlc* promoter (−719 to −1114), where self-activation precluded use of the β-gal assay and interactions were detected by growth in the presence of 3-aminotriazole. Colonies were arrayed in quadruplicates to aid identification of positives. Numbers indicate start and end of fragments relative to the gene's start codon in the genome. (D) Comparison of MITOMI and yeast one-hybrid results. Promoter fragments are depicted by grey and green horizontal bars, with start codon to the right. Fragments displaying interaction with Her1 are in green, and fragments without interaction with Her1 are in grey. Vertical bars indicate H-box sites. Relative binding affinity of each H-box to Her1 as determined by MITOMI is color-coded from black (low affinity) to yellow (high affinity). For relative affinities of individual sites, see Table S1. Numbers indicate distance from start codon. (E) Bait strains were transformed with pAD-DIMER vectors encoding two copies of AD-tagged *her1*, *her7*, or *hes6* or a combination of *her7* and *hes6* and interactions detected as in (A–C). Only one fragment each from the *her1*, *her7*, and the *dlc* promoter gives signals with Her1 only expressed from pAD-DIMER, and the same fragments interact with a combination of Her7 and Hes6.

To validate our findings from these biochemical assays in a more physiological setting, we employed a yeast one-hybrid (Y1H) assay to assess binding of Her proteins to cyclic gene promoter fragments in the context of a eukaryotic nucleus. We selected 18 promoter fragments from approximately 100 bp to 1 Kb in length that cover between 3 and 4 Kb upstream of the transcriptional start sites of *her1*, *her7*, and *dlc*, cloned them upstream of the lacZ and the His3 reporter genes and stably integrated them into the yeast genome. We first used the pDEST_AD_ vector system [24],[25] to express individual Hes/Her proteins tagged with the Gal4 activation domain (AD) as protein prey in our DNA bait strains. In this assay we found interaction of Her1, but not Her7 or Hes6, with a number of DNA baits (Figure 3A–C). This is consistent with the results of our MITOMI assays and confirms that Her1 homodimers, but not Her7 or Hes6 homodimers, have DNA binding activity. Seven of the nine fragments that displayed interactions with Her1 in this Y1H assay contain H-box sites that showed medium or high affinity binding to Her1 in the MITOMI assay (red or yellow bars in Figure 3D), whereas the majority of fragments that were negative in the Y1H contain no H-box sites or H-box sites with low affinity (black bars in Figure 3D). In the two fragments from the *dlc* and the *her7* promoter that contain a high-affinity H-box site but do not give a signal in this assay, steric factors such as nucleosome arrangement might hinder transcription factor binding or reporter gene expression. Taken together, these Y1H assays suggest that most of the binding sites we identified in vitro are bound by Her proteins in the context of a eukaryotic nucleus.

Next, to confirm our finding that Her7 and Hes6 gain DNA binding activity through heterodimerization, we used a novel Gateway-compatible vector called pDEST_AD-DIMER_ (Hens et al., in preparation) that allows expression of two AD-fused proteins from the same vector. This vector system has lower sensitivity than the pDEST_AD_-system—when we expressed two copies of Her1 from the pDEST_AD-DIMER_ vector we detected only three interacting fragments, those that gave strong signals with the pDEST_AD_-system (compare Figure 3E to Figure 3A–C). Expression of two copies of Her7 or Hes6 gave no signal, but co-expression of Her7 and Hes6 resulted in interaction with the same fragments that were targeted by Her1 (Figure 3E). This finding is consistent with our in vitro measurements and suggests that, in the context of a eukaryotic cell, Her7 and Hes6 need to heterodimerize to gain DNA binding activity, and that Her1 homodimers and Her7:Hes6 heterodimers target the same sites in the promoters of the cyclic genes *her1*, *her7*, and *dlc*. Together, the protein–DNA binding assays described here suggest a network topology for transcriptional regulation in the segmentation clock where Her1 homodimers and Her7:Hes6 heterodimers form two parallel redundant feedback loops that converge on the same regulatory sites in the *her1*, *her7*, and *dlc* promoters.

### Oscillatory Gene Expression and Segmentation Is Maintained in the Absence of *hes6* or *her1*, But Not *her7*

To assess the relevance of the two-loop network described above for oscillatory gene expression and segmentation in the embryo, we decided to examine the phenotypes of embryos with mutations in the network's components. Because each of the three genes is involved in only one of the two feedback loops, the two-loop model predicts that *her1*, *her7*, and *hes6* single gene mutants should all be competent to oscillate and to support somite segmentation.

The *hes6* mutant has previously been shown to segment normally along its entire axis [17], and accordingly we found clear evidence for transcriptional oscillations of *her1*, *her7*, and *dlc* at the 10-somite stage (Figure S3A–C). The *hes6* mutant phenotype is therefore in agreement with the predictions of the network topology. Previously reported defects in cyclic gene oscillations after *hes6* morpholino knockdown may have resulted from off-target effects, or from the temperature at which the embryos were incubated in these experiments [18],[26].

The consequences of loss of *her1* and *her7* function in somitogenesis have also previously been addressed using morpholino antisense oligonucleotides [11],[12],[27]. To overcome possible off-target effects or incomplete gene knockdown associated with this approach, we decided to re-examine *her1* and *her7* loss-of-function phenotypes in genetic mutants. These mutants were generated by ENU mutagenesis [28] and carry premature stop codons in the *her1* and *her7* genes, respectively (Figure S4A,B,E,F). These genetic lesions lead to full loss of function of the respective gene (Figure S4C,D,G,H).

**Figure 4:**
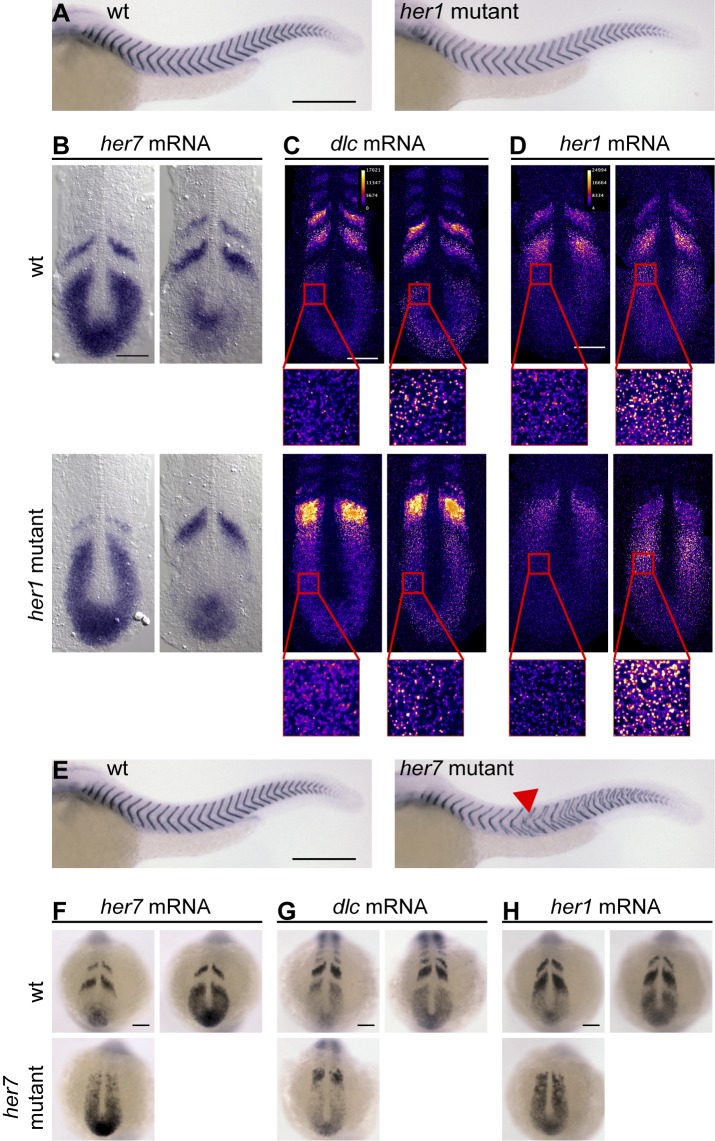
Genetic mutants indicate that *her7* and *her1* have distinct functions. (A) Wildtype (wt, left) and *her1* mutant (right) embryos at 34 hours post fertilization (hpf) stained for *cb1045* expression to visualize segmentation. Anterior segmentation defects in *her1* mutant embryos were of variable severity, and a representative embryo is shown. See Figure S4D for a quantitative analysis of segmentation defects in *her1* mutants. (B–D) wt (upper row) and *her1* mutant (lower row) embryos at the 10-somite stage in situ stained for *her7* (B), *dlc* (C), or *her1* (D) mRNA expression. *her1* and *dlc* expression patterns (C and D) were visualized using tyramide chemistry and displayed in FIRE lookup table to distinguish onset of expression waves (high intensity punctate signal in insets) from later phases of the oscillation cycle (punctae with lower intensity or diffuse signal). (E) wt (left) and *her7* mutant (right) embryos at 34 hpf stained for *cb1045* expression to visualize segmentation. Red arrowhead points to anterior-most segmentation defects in *her7* mutants. (F–H) wt (upper row) and *her7* mutant (lower row) embryos at the 10-somite stage in situ stained for *her7* (F), *dlc* (G), or *her1* (H) mRNA expression. (A) and (E–H) are whole mount preparations, and (B–D) are flat mount preparations. (B–D) and (F–H) Two representative examples per condition are shown to illustrate oscillatory expression, except for *her7* mutants (F–H). Scale bar: 300 µm for (A and E), and 100 µm for all other panels.

Most *her1* mutants segmented normally along most of the trunk and tail (Figure 4A). Segmentation defects occurred only in a subset of *her1* mutant embryos and were preferentially localized in the anterior trunk (see Figure S4C and D for a quantitative analysis of segmentation defects in *her1* mutants). We observed evidence of tissue-level transcriptional oscillations in *her1* mutants already at the bud stage (Figure S5A,B), suggesting that the partially penetrant anterior defects are not caused by a failure in establishing transcriptional oscillations at early somitogenesis stages. Tissue-level transcriptional oscillations of *her7* mRNA were also evident at the 10-somite stage (Figure 4B). Because the wave pattern of transcriptional oscillations is altered in *her1* mutants compared to wildtype embryos, it is difficult to visualize oscillating expression of *her1* and *dlc* mRNA on the tissue level using standard chromogenic reagents. Subcellularly, transcriptional oscillations manifest in a succession of distinct localizations of the mRNA of oscillating genes—in early phases of the oscillatory cycle, cyclically expressed mRNAs are found in distinct nuclear spots at the sites of transcription, whereas later in the cycle they localize to the cytoplasm and give a diffuse staining [29]. We therefore used tyramide chemistry to detect changes in the subcellular localization of mRNA as a proxy for transcriptional oscillations. With this method, we were able to detect both *her1* and *dlc* mRNA in distinct subcellular localizations in different *her1* mutant embryos (Figure 4C,D), indicating that oscillations of *dlc* mRNA and the mutant *her1* mRNA continue in the *her1* mutant PSM. The *her1* mutant phenotype is therefore in agreement with the predictions from the two-loop network topology—in the absence of Her1, the function of the Her7:Hes6 heterodimer is sufficient to drive oscillatory expression of *her7*, *dlc*, and the mutant *her1* mRNA. The genetic *her1* loss-of-function phenotype described here is consistent with reports from previous studies using MO-mediated gene knockdown [11],[12],[30] and suggests that the more widespread segmentation defects upon *her1* knockdown that were reported by one other study [27] reflect off-target effects.

*her7* mutants, in contrast to *her1* and *hes6* mutants, patterned only the anterior trunk correctly, and segmentation became defective in all *her7* mutant embryos at the level of the 10^th^ or 11^th^ segment (Figure 4E, red arrowhead; Figure S4G,H). These posterior segmentation defects are accompanied by the decay of tissue-level transcriptional oscillations of *her1*, *her7*, and *dlc* between the bud- and the 10-somite stage (Figure 4F–H, Figure S5C–E). This *her7* mutant phenotype is consistent with previous studies using MO-mediated *her7* knockdown, where tissue-level oscillations have been shown to decay gradually [11],[12],[30]. The oscillatory behavior in the anterior trunk of *her7* mutants is consistent with our expectations, but the highly penetrant posterior failure of cyclic gene expression and segmentation is not predicted by the simple two-loop network topology. The finding that posterior segmentation defects are observed in *her7* but not *hes6* mutants suggests that the Her7:Hes6 heterodimer is not the only functional species involving these proteins. We will return to this issue below.

### *her1*, *her7*, and *hes6* Are Essential Components of the Zebrafish Segmentation Clock

The observation that transcriptional oscillations occur in the absence of *her1*, *her7*, or *hes6* alone supports the two-loop topology determined in our protein-DNA binding studies. A further prediction from this topology is that lesions to both loops should cripple the oscillator. To test this idea, we analyzed the phenotypes resulting from combined loss of *her1* and *her7* or *her1* and *hes6* function. In agreement with previous observations using only MO-mediated gene knock-down [11],[12],[31] we found that *her1;hes6* double mutants and *her1* mutants injected with *her7* targeted MOs displayed a failure of segmentation along the entire axis (Figure S6A,C) and show no sign of oscillatory gene expression at the 10-somite or bud stage, respectively (Figure S6B,D). These findings indicate that there are no feedback loops in the zebrafish segmentation clock that operate independently from *her1* or *her7* and *hes6* and that are sufficient to drive tissue-level transcriptional oscillations of *her1*, *her7*, or *dlc*, or segmentation of the embryo's body axis. Importantly, since lesions in both of the predicted loops prevent cyclic gene oscillations and embryonic segmentation, these phenotypes are again in agreement with the proposed network topology.

### Mutating *hes6* Alleviates Posterior Segmentation Defects Caused by Loss of *her7*

**Figure 5:**
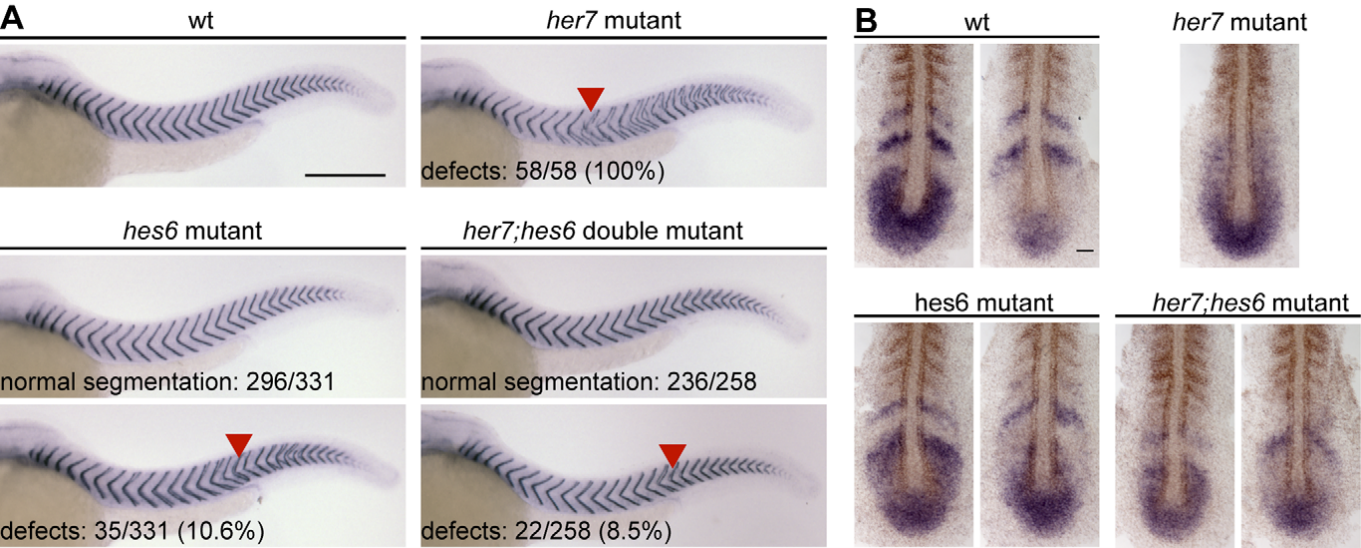
The *hes6* mutant phenotype is epistatic to the *her7* mutant phenotype. (A) Wildtype (wt, upper left), *her7* mutant (upper right), *hes6* mutant (lower left), and *her7;hes6* double mutant embryos (lower right) at 34 hpf stained for *cb1045* expression to analyze segmentation. Posterior segmentation defects (red arrowheads) are fully penetrant in *her7* mutants, but not in *hes6* mutant and *her7;hes6* double mutant embryos. Numbers indicate number of embryos with and without segmentation defects for *hes6* and *her7;hes6* mutants. Whole mount preparations, anterior to the left, scale bar 300 µm. (B) wt (upper left), *her7* mutant (upper right), *hes6* mutant (lower left), and *her7;hes6* double mutant embryos (lower right) at the 10-somite stage in situ stained for *her7* expression (blue). Formed segments are labeled by staining for *myoD* mRNA expression (brown). Two representative examples with different expression patterns are shown for wt, *hes6*, and *her7;hes6* mutants to indicate evidence for ongoing tissue-level oscillations. Patterns in all *her7* mutants examined were similar, and one representative example is shown. Flat mount preparations, anterior to the top, scale bar 50 µm.

As a last test of the network's two-loop topology we examined cyclic gene expression and segmentation in *her7*;*hes6* double mutants. Since Her1 homodimers with DNA binding activity are available in this condition, the network structure predicts that oscillations will at least initiate in this double mutant. Yet because of the role of Her7 in maintaining tissue-level oscillations throughout segmentation stages that became apparent in the *her7* single mutant, we expected the oscillations to likewise decay in *her7*;*hes6* mutants, and segmentation to become strongly defective in the posterior trunk and tail. However, we found that most *her7;hes6* double mutants segmented normally and posterior segmentation defects occurred with a low severity and penetrance comparable to *hes6* single mutants (Figure 5A). Furthermore, the expression patterns of the mutant *her7* mRNA in *her7;hes6* double mutants at the 10-somite stage showed clear indications of tissue-level oscillations, similar to the situation in *hes6* single mutants (Figure 5B). These results indicate that *hes6* is fully epistatic over *her7*: Since identical phenotypes are observed in *hes6* single and *her7;hes6* double mutants, *her7* function in somitogenesis is entirely dependent on the presence of *hes6*. On the other hand, the fact that the decay of oscillatory gene expression and defective segmentation in a *her7* mutant background can be rescued by mutating *hes6* indicates that *hes6* is not neutral in the absence of *her7* function, but dominantly interferes with clock function in this condition. This suggests that segmentation defects in *her7* mutants are caused by Hes6 protein, which would contribute to Her7:Hes6 heterodimers in wildtype embryos, but in the absence of Her7 interferes with other critical components of the segmentation clock, such as Her1 homodimers. Importantly, the normal segmentation and cyclic gene expression patterns in the *her7*;*hes6* double mutant combination is in agreement with the redundant two-loop negative feedback network topology predicted by our in vitro studies.

We conclude that the single and double mutant phenotypes support the expectations from the biochemically determined two-loop topology of the segmentation clock's core circuit, but also reveal additional functions of Her7 and Hes6 in the segmentation clock.

### Loss of *her1* or *her7* Function Does Not Alter Somitogenesis Period

While the phenotypic assays described above provide a straightforward test of the circuit's basic topology by asking whether the oscillatory state of the circuit can be initiated and maintained in mutant conditions, they do not probe more subtle aspects of the circuit's dynamics. In particular, neither the static phenotypes nor the simple topology of the network allow inference of how the period of oscillations is regulated. A previous mathematical model of the segmentation clock has emphasized the role of transcriptional and translational delays in a *hes/her* feedback system in setting the period of oscillations [10]. Specifically, it would be predicted that oscillations relying exclusively on a *her7*-based feedback loop might be faster than oscillations that are exclusively based on *her1*, because of shorter production delays in the *her7*-based loop. We have previously shown that mutating *hes6* slows segmentation clock period [17], and our protein-DNA binding data indicate that Her7 requires Hes6 to gain DNA binding activity. Therefore, it seemed possible that the period slowing in *hes6* mutants was due to the loss of the fast Her7:Hes6 loop and reflected a slower Her1-based loop operating in isolation. If this were the case, then we would expect that the formation of anterior segments in *her7* single and *her7;hes6* double mutants would be slowed as in *hes6* mutants. Furthermore, if the slower *her1* loop modulated the period in the wildtype, *her1* mutants might segment faster than wildtype embryos.

**Figure 6:**
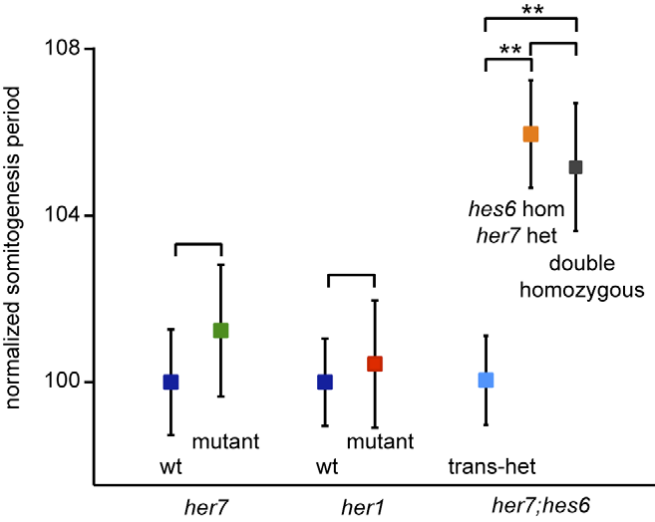
Somitogenesis period in *her1*, *her7* single and *her7;hes6* double mutants. Somitogenesis period was measured by multiple-embryo time-lapse microscopy. Measurements in single mutants were carried out using incrosses from heterozygous carriers, measurements in *her7* heterozygous; *hes6* homozygous and *her7;hes6* double homozygous mutants were performed with embryos obtained from crosses of trans-heterozygous and double homozygous carriers. Wildtype (wt) siblings were used as controls for single mutants, and trans-heterozygous siblings were used as controls for *her7;hes6* double mutants. Analysis was performed by an observer blind to the embryos' genotype, and measurements of somitogenesis period from individual embryos were normalized to the mean of the somitogenesis period of control embryos in the experiment. Data show mean values pooled from three separate experiments per genotype, and error bars indicate 95% confidence intervals, *n*≥25 for each genotype or control. ** indicates *p*<0.01 by Mann-Whitney *U*-test, two-tailed.

We used multiple-embryo time-lapse imaging to test this idea and recorded the periodicity of somite formation as a morphological proxy for the oscillations of the segmentation clock [32],[33]. We found that somites form with similar dynamics in wildtype, *her1* mutant, and *her7* mutant embryos (Figure 6). Somitogenesis period in *her7;hes6* double mutants was slowed compared to trans-heterozygous controls, and comparable to *hes6* homozgyous;*her7* heterozygous mutants (Figure 6). These findings indicate that neither *her1* nor *her7* have a crucial role in setting the tempo of segmentation clock oscillations, and that *hes6* regulates segmentation clock period independently from simply providing a heterodimerization partner for Her7. The period control function of *hes6* can therefore not be mapped to the simple two-loop topology determined by our protein-DNA interaction studies, and this motivated us to consider a possible role for non-DNA binding dimers in regulating the dynamics of the segmentation clock.

### Hes/Her Protein-DNA and Protein-Protein Interactions Are Sufficient to Explain the Observed Embryonic Phenotypes

**Figure 7:**
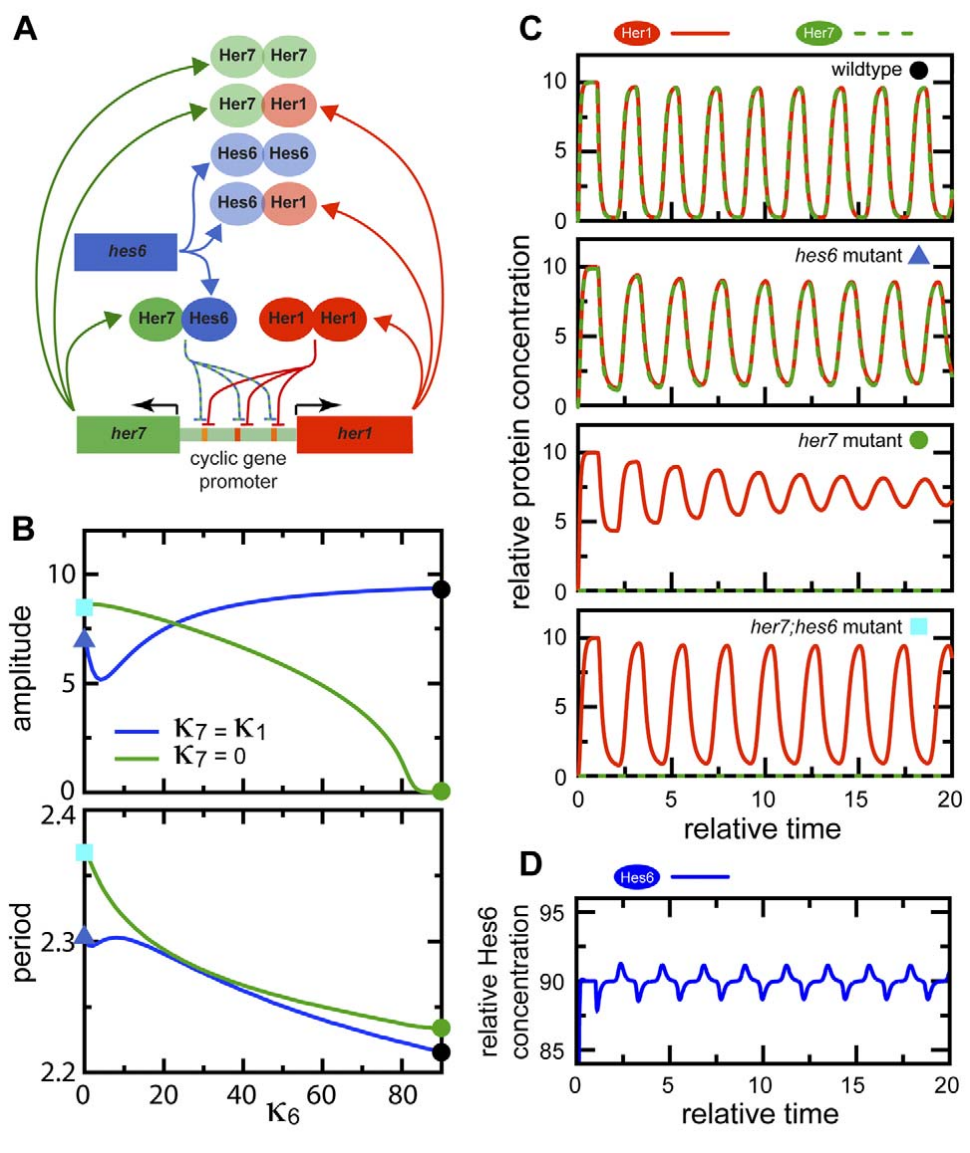
A data-based mathematical model of the clock's core circuit can recapitulate mutant dynamics. (A) Schematic representation of experimentally determined protein-protein interactions and protein-DNA interactions, neglecting weak protein-DNA interactions. Proteins are represented as ovals and genes by rectangular boxes. Blunted arrows represent repression of *her1* and *her7* promoters by Her1 homodimers and Her7:Hes6 heterodimers. Colored arrows represent production. (B) Amplitude (maximum minus minimum concentration of total Her1 protein at steady state) and period of oscillations obtained from numerical simulations of the minimal model (Materials and Methods and Text S1), plotted as a function of the scaled production rate of Hes6 protein *κ*_6_, for the two situations *κ*_7_ = *κ*_1_ (blue line) and *κ*_7_ = 0 (green line). The symbols mark different situations that qualitatively describe mutant conditions: *hes6* mutant (blue triangle), *her7* mutant (green dot), *her7;hes6* mutant (cyan square), and wildtype (black dot). Parameters: *τ*_1_ = 1.02, *τ*_7_ = 1.00, *κ*_1_ = 10.0, *δ* = 1.0, and *n* = 2.0. (C) Illustration of Her1 and Her7 dynamics for different situations marked in (B), recapitulating features observed in mutant phenotypes. (D) Illustration of Hes6 dynamics in wildtype. Although Hes6 production is constant in time, Hes6 levels oscillate due to rhythmic changes in effective stability.

To explore the complex regulatory possibilities of the circuit we have described and determine whether wildtype and mutant phenotypes can be explained in a rigorous, internally consistent manner, we decided to investigate the dynamics of a mathematical model of the network describing the behavior of Her1, Hes6, and Her7 proteins (see Text S1, Tables S2, S3, and Figures S7, S8, S9, S10, S11 for details). Following our protein-protein and protein-DNA binding results, our model allows for formation of all possible dimers between Her1, Hes6, and Her7, but negative feedback regulation of *her1* and *her7* occurs exclusively through Her1:Her1 homodimers and Her7:Hes6 heterodimers (schematically depicted in Figure 7A). We use a Hill function to describe repression through these dimers in our model. This is motivated by our finding that there are multiple binding sites for each of the dimers in every cyclic gene (Figure 3D) [34],[35] and does not reflect any assumptions about the binding mechanism of Hes/Her dimers to individual target sites. For simplicity, we ignore potential transcriptional regulation via dimers with weak DNA binding activity. Nevertheless, the dimers that do not bind DNA in our model still perform an important post-translational regulatory function, since they sequester monomers and thereby affect the availability of dimers with DNA binding activity. The period measurements in Figure 6 provide strong constraints on the network's dynamics, and can be used to guide the choice of parameter values.

To understand the consequences of the basic network topology, and because reliable measurements of the rate constants of the processes in this network are not available, we intentionally avoid automatic methods to optimize model parameters to obtain the best possible quantitative fit to experimental results. Instead, when possible we keep rate constants associated with production, dimerization, and repression equal between the Hes/Her proteins (Table S3), and introduce only the minimum differences between species necessary to qualitatively reproduce experimental trends. For this reason, the parameter values we report below should not be understood as exact quantitative predictions. To simplify the model as far as possible, we focus only on the generation of oscillations within single cells and do not describe cell-cell coupling.

To analyze the dynamics of this network, we obtained numerical solutions of the minimal version of the model. The different mutant conditions were simulated by setting the production rate of the corresponding component(s) to zero. We started by setting the Hes6 production rate to zero, *κ*_6_ = 0, to describe a *hes6* mutant; in this situation, the network can support high-amplitude oscillations (blue triangle in Figure 7B and second panel in Figure 7C). As the value of *κ*_6_ is increased, the period of oscillations decreases (blue line in Figure 7B, bottom; Figure S7). At *κ*_6_ = 90, the period of the simulated oscillations is approximately 6% faster than in a situation without *hes6* (black point in Figure 7B), matching the experimentally observed difference in somitogenesis period between *hes6* mutant and wildtype embryos (Figure 6C) [17]. We therefore chose *κ*_6_ = 90 as the wildtype value for Hes6 production. This value for Hes6 production is higher than the corresponding values *κ*_1_ = *κ*_7_ = 10 for Her1 and Her7 production, thereby distinguishing Hes6 from Her1 and Her7 at the parameter level. A parameter sensitivity analysis shows that the period and amplitude of oscillations are robust to changes in most of the parameters of the model (Figure S8).

This dependence of oscillation period on Hes6 production rate is caused by heterodimerization of Hes6 with Her1 and Her7, allowing Her1 and Her7 proteins to be degraded also as components of these heterodimers, which alters the effective stability of Her1 and Her7 (Figure S9). A striking consequence of the regulation of the effective half-lives of Her1 and Her7 by dimerization with Hes6 in the model is that Hes6 protein levels also oscillate, albeit with relatively low amplitude, despite a constant production (Figure 7D). We return to this distinctive prediction below.

Next, we simulated the *her7* mutant by setting the Her7 production rate to zero, *κ*_7_ = 0. We find that the period of these simulated oscillations is shorter for a *her7* mutant than the wildtype over a range of values for *κ*_6_ (Figure S7), in contrast to our experimental measurements (Figure 6). To fit our experimental data with our model as simply as possible, we introduce one more parameter asymmetry, choosing a slightly longer production delay *τ* for Her1 compared to Her7. Qualitatively, this choice appears to be justified by the physical properties of the two genes, because the *her1* gene is longer and contains more introns than the *her7* gene. Quantitatively, a relatively small difference is motivated from independent experimental data, because transcription is fast (∼4 Kb/min), intron splicing is co-transcriptional, and splicing times are short (∼5 min) and independent of length [36]. For a difference of 2% between the *her1* and *her7* delays, the oscillations in the simulated *her7* mutant have a period similar to the wildtype situation, almost independently of our choice for *κ*_6_ (green line in Figure 7B). At *κ*_6_ = 90, oscillations initiate in the simulated *her7* mutant, but the amplitude of these initial oscillations decreases over time, eventually falling to zero (green dot in Figure 7B, Figure 7C). This suggests that in the embryo, mutation of *her7* results in lower amplitude or even fully damped oscillations on the single cell level, and this gradual damping of oscillations provides an explanation for the posterior segmentation defects in *her7* mutants.

While the amplitude of oscillations in the simulated *her7* mutant decays instantaneously and rapidly and Her7 trough levels are constantly elevated in this situation (Figure 7C), the amplitude of the early oscillations in bud stage embryos appears to be similar to the wildtype (Figure S5C–E). Therefore, although our model is qualitatively successful in explaining the *her7* mutant phenotype, there remains a quantitative difference between the model and the data. We speculate that this may be due to the particular choice of parameters in our model, which have not been optimized to capture the amplitude of oscillations in early *her7* mutant embryos, or due to effects of coupling between cells on the tissue level, which are not represented in our model.

In our model, the reduction of amplitude in the *her7* mutant simulation arises because the resulting level of Her1 homodimer is insufficient to sustain oscillations (Figure S10). Although Her1 monomer production increases due to loss of repression via Her7:Hes6 heterodimers, most of it is sequestered as non-DNA binding Her1:Hes6 heterodimers. Consequently, decreasing *κ*_6_ in a simulated *her7* mutant situation leads to a recovery of the amplitude of oscillations (green line in Figure 7B). Our model thereby also provides an explanation for the striking rescue of segmentation defects in the *her7;hes6* double mutant. The period of oscillations in *her7;hes6* double mutant simulations is slowed compared to the wildtype situation, in qualitative agreement with our experimental findings. We note, however, that our model predicts a period difference between the *hes6* single and *her7;hes6* double mutant situation (cyan square in Figure 7B and Figure 7C, bottom), which is not observed experimentally (Figure 6). In the model, this period difference is caused by heterodimerization and effective destabilization of Her1 by Her7 in the *hes6* single, but not in the *her7;hes6* double mutant. The difference between model and experiment suggests that we either overestimate this destabilization effect in our minimal parameterization of the model by setting all rate constants equal or that other proteins in the PSM that are not considered in this model can compensate for Her7 function in regulating Her1 stability.

In summary, our mathematical model indicates that the two-loop negative feedback topology of the *her* gene network as determined by our protein-protein and protein-DNA interaction studies is sufficient to explain the qualitative dynamic characteristics of the different experimentally observed single and double mutant phenotypes when the effects of dimers with and without DNA binding activity are considered. The success of this model supports an important role for both types of dimers in controlling the dynamics of the circuit, since together they determine the availability of DNA binding dimers. Below, we report on three experimental tests of the formation of Hes/Her dimers described in the model.

### *hes6* Dosage Affects Clock Function In Vivo

Our mathematical model makes predictions about the role of Hes6 production rate in controlling the period and the amplitude of oscillations. We reasoned that the expected roles of Hes6 production rate should be testable using the *hes6* heterozygote mutant embryo. As predicted by our model, we observed that the periodicity of somite formation in *hes6* heterozygous mutant embryos was slowed compared to their wildtype siblings (Figure S12A) and leads to a reduction in segment number that is in agreement with the period measurements (Figure S12B,C). Furthermore, we found a shift of the onset of defects towards the posterior when the *hes6* locus was heterozygous mutant in a *her7* mutant background, compared to *her7* mutants with two wildtype *hes6* loci (Figure S12D,E). This is consistent with the predicted tuning of the amplitude of Her1 oscillations by Hes6 levels. Together, these findings from *hes6* heterozygous mutants are in agreement with the predictions of our mathematical model, providing additional support for the idea that Hes6 quantitatively affects segmentation clock functions by titrating critical oscillatory monomers and affecting their effective stability.

### Hes6 Protein Oscillations

**Figure 8:**
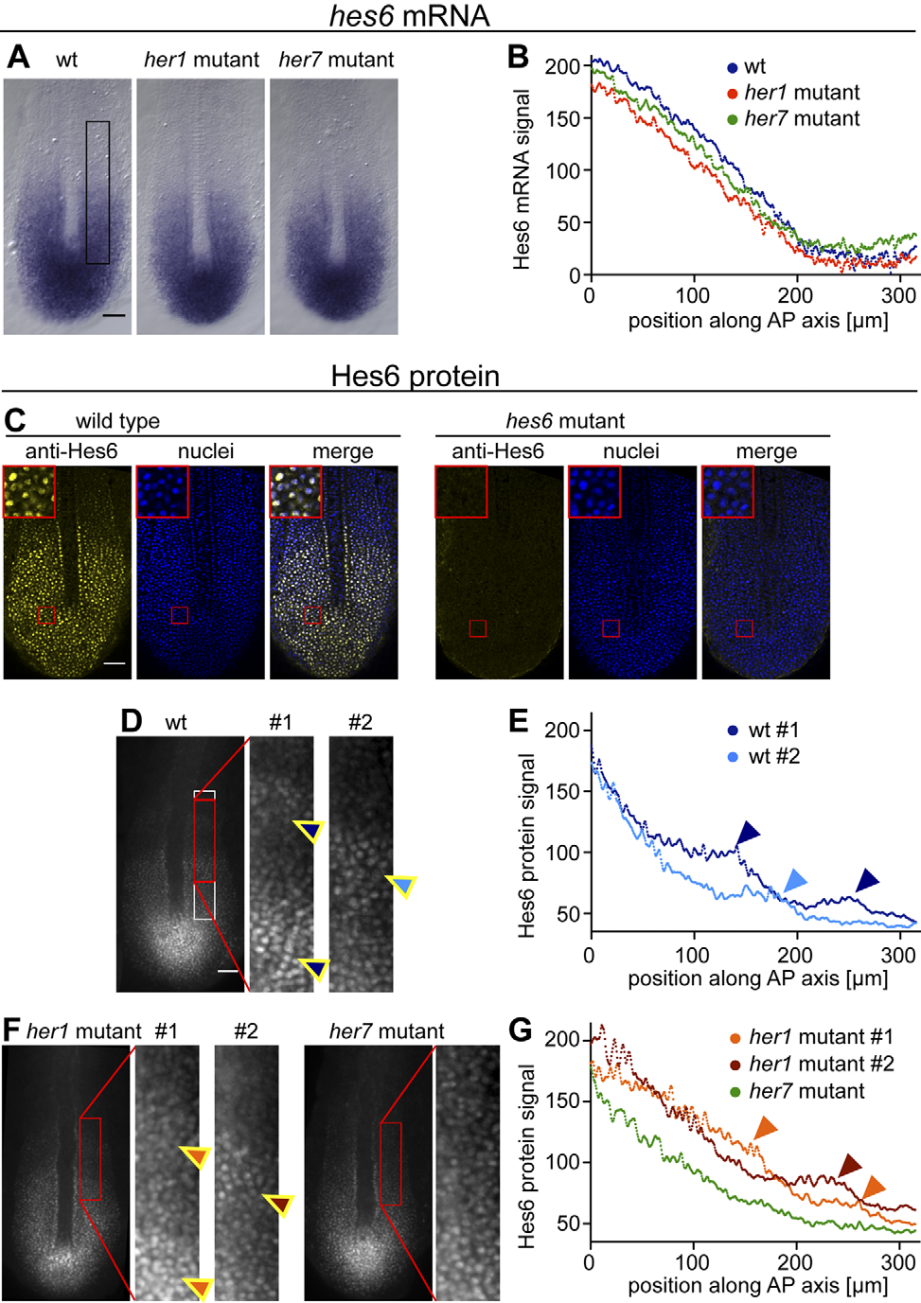
Posttranscriptional Hes6 protein oscillations. (A) Wildtype (wt, left), *her1* mutant (middle), and *her7* mutant embryos at the 10-somite stage in situ stained for *hes6* mRNA expression. (B) Intensity profile of *hes6* mRNA staining in the embryos shown in (A), using the region boxed in black in the rightmost panel in (A). The origin of the graph corresponds to the end of the notochord. *hes6* mRNA levels decay smoothly from posterior to anterior in wildtype, *her1*, and *her7* mutant embryos. (C) Single confocal sections of 10-somite stage wildtype (left) and *hes6* mutant embryos (right) immunostained with monoclonal antibodies raised against full-length Hes6 (yellow). Nuclei are counterstained with Hoechst 33342 (blue). Antibodies show immunoreactivity with a nuclear antigen in wildtype, but not in *hes6* mutant embryos. Insets show magnified view of region boxed in red in the tailbud. (D) Widefield images of wildtype embryos at the 10-somite stage immunostained with Hes6 antibodies. Dynamic waves of Hes6 staining in the intermediate PSM (arrowheads) become apparent upon enhancing the contrast in this region (panels to the right). (E) Intensity profile of Hes6 protein staining for the embryos shown in (D) using the region boxed in white in the rightmost panel of (D). The origin of the graph corresponds to the end of the notochord. Waves of Hes6 protein levels manifest as local peaks in the staining profile (arrowheads). (F) Widefield images of *her1* mutant (left) and *her7* mutant (right) embryos at the 10-somite stage immunostained with Hes6 antibodies. Waves of Hes6 staining in the intermediate PSM can be observed upon contrast enhancement in *her1* mutant embryos (arrowheads), but not in *her7* mutant embryos. (G) Intensity profiles of Hes6 protein staining for the embryos shown in (F). Arrowheads point towards local peaks in the intensity trace indicative of protein oscillations, which can be detected for *her1* mutant embryos (light and dark red traces), but not for the *her7* mutant embryo (green trace). (A, C, D, F) Flat mount preparations, anterior to the top; scale bars, 50 µm.

Finally, we sought to directly investigate the predicted effects of dimerization on the effective protein stability of Hes6. Our model suggests that dimerization of Hes6 with the cyclically expressed proteins Her1 and Her7 and subsequent degradation of the Her1:Hes6 and Her7:Hes6 heterodimers will manifest in post-transcriptional oscillation of Hes6 protein levels (Figure 7D), and we decided to test this prediction directly in the embryo (Figure 8). To first rule out that any potential oscillatory protein pattern could be caused by transcriptional oscillations of *hes6* mRNA that had previously been overlooked [5],[17],[18], we searched 5 Kb upstream of the *hes6* start codon for H-box sites. The only H-box-containing 12-mer within this stretch of DNA has the sequence ACTCACGTGAGA (unpublished data). Because the central CACGTG consensus is flanked by T and A, respectively, this H-box is not expected to be strongly bound by Her1 homodimers or Her7:Hes6 heterodimers (Table S1). Furthermore, when we examined the spatial pattern of *hes6* mRNA expression in wildtype embryos at the 10-somite stage, we observed a smooth decay of the staining intensity from posterior to anterior; there was no evidence for spatial mRNA waves, which would be expected if Her1 or Her7 controlled *hes6* expression (Figure 8A,B, *n* = 20). Finally, the pattern of *hes6* mRNA expression is not altered in *her1* and *her7* mutants (Figure 8A,B, *n*≥20 for each genotype). This indicates that *hes6* mRNA expression is not subject to transcriptional control by Her1 or Her7. Taken together, these data indicate that *hes6* mRNA levels do not oscillate, in line with reports from several previous studies [5],[17],[18].

To test the prediction that Hes6 protein levels oscillate, we raised monoclonal antibodies against Hes6. Whole-mount immunostaining revealed a nuclear signal in the tailbud and posterior PSM of wildtype embryos (Figure 8C, left). No nuclear signal was obtained in *hes6* mutant embryos, indicating the specific detection of Hes6 protein (Figure 8C, right). When we examined Hes6 immunoreactivity in wildtype embryos, a subset (10/26) showed striped staining patterns indicative of Hes6 protein oscillations (arrowheads in Figure 8D). These patterns can be compared by plotting the intensity profile of Hes6 immunoreactivity in the intermediate PSM (Figure 8E). Their shape and position recapitulates the wave pattern of *her1* and *her7* mRNA expression in this region of the PSM. Although we cannot formally rule out the possibility that these Hes6 protein oscillations might be produced by *hes6* mRNA oscillations below our detection limit or by an influence of cyclically expressed proteins on Hes6 translation, this finding is consistent with our prediction that the cyclically expressed proteins Her1 and Her7 regulate Hes6 protein stability in the PSM.

To test the influence of Her1 and Her7 proteins on Hes6 protein oscillations, we examined Hes6 immunoreactivity in *her1* and *her7* mutants. If *Her1* and *Her7* oscillations were responsible for generating the tissue-level Hes6 protein oscillations, they should be lost in *her7* mutants, where tissue-level oscillations of all cyclic genes examined decay by the 10-somite stage (Figure 4F–H). In contrast, Hes6 protein oscillations should remain in *her1* mutants, where *her7* is still expressed in a wave pattern (Figure 4B). In *her1* mutants, we detected anterior waves of Hes6 immunoreactivity (*n* = 4/16, red arrowheads in Figure 8F,G), in line with our expectation of ongoing Hes6 protein oscillations in *her1* mutants. These patterns were less pronounced than in wildtype embryos, consistent with the altered wave-like expression patterns of *her7* mRNA in the *her1* mutant (Figure 4B,D). In contrast to wildtype and *her1* mutant embryos, we found no evidence for tissue-level protein oscillations in *her7* mutants (Figure 8F,G, *n* = 16). Hes6 protein levels decayed smoothly from posterior to anterior, in parallel with the shape of the *hes6* mRNA profile (Figure 8A,B,F,G), in line with our expectation. Taken together, our finding of characteristic cyclic wave patterns of Hes6 protein in wildtype embryos (despite smooth *hes6* mRNA patterns) and the differentially perturbed patterns of Hes6 protein oscillation in the *her1* and *her7* mutants is consistent with one of the key features of our model, namely that heterodimerization regulates effective protein stability in the segmentation clock.

## Discussion

In this work we describe a new dynamic model for the core pace-making circuit of the segmentation clock, which is based on a systematic biochemical investigation of the network's topology and precise measurements of somitogenesis dynamics in novel genetic mutants. Our key finding is that the core pace-making circuit consists of two distinct negative feedback loops, one involving Her1 homodimers and the other involving Her7:Hes6 heterodimers, operating in parallel. We can account for the single and double mutant phenotypes of *her1*, *her7*, and *hes6* mutants in a mathematical model of this core circuit, wherein protein-protein interactions between oscillating and non-oscillating Hes/Her genes control the availability of dimers with DNA binding activity. This is a new principle for the regulation of oscillatory dynamics that may be relevant beyond the zebrafish segmentation clock.

### A Novel Topology for the Segmentation Clock's Core Circuit

The protein-DNA interaction assays in this article extend previous efforts to biochemically map regulatory interactions in the segmentation clock. The interaction of Her1 homodimers with the *her1* promoter using electrophoretic mobility shift assays was previously observed [14]. While this article was under review, evidence for the existence of binding sites for Her1 homodimers and Her7:Hes6 heterodimers in the *her7* promoter was published [26]. Here we show that, in addition, Her7:Hes6 heterodimers also bind to the *her1* promoter, and both Her1 homodimers and Her7:Hes6 heterodimers target sequences in the *dlc* promoter. Using MITOMI technology, we were able to determine and compare the DNA binding specificity of both types of dimers in an exhaustive and unbiased way. We find that both dimers bind to the core consensus sequence CACGNG. This sequence differs from the N-box sequence CAC[G/A]AG commonly reported for mouse Hes proteins [37], which had been determined via a candidate approach, but is similar to the consensus binding site CACG[T/C]G established for the *Drosophila* Hairy protein [38]. Therefore, it appears likely that binding to the CACGNG consensus sequence is a common feature of Hairy-related bHLH transcription factors in different species, and we term this consensus binding site the H-box. When we compare the relative affinities of Her1 homodimers and Her7:Hes6 heterodimers to H-box sites in the three cyclic gene promoters, we detect differences. Although this observation is in line with recent results [26], our data do not support the proposed hypothesis [26] that these differences might have functional consequences to the dimers in the context of the segmentation clock, since sites with relatively higher affinity to either of the two types of dimer do not appear to be enriched in any of the three promoters examined.

Our protein-DNA interaction experiments suggest a regulatory network architecture for the segmentation clock where *her1* and *her7* engage in two parallel, redundant negative feedback loops, which converge on the same DNA regulatory elements (Figure 7A). The concept of redundant *her1*- and *her7*-based feedback loops has been introduced before in dynamic models of the segmentation clock, following genetic evidence [8],[10],[11]. However, the biochemical realization of these redundant feedback loops that we determine here differs from these earlier models. Previously, all possible dimers between Hes/Her proteins were assumed to have DNA binding activity and participate in feedback regulation. We find here that although Hes/Her proteins indeed dimerize promiscuously, only Her1 homodimers and Her7:Hes6 heterodimers have strong DNA binding activity. These findings are again in general agreement with the recent study mentioned above [26]. An equilibrium description of the Hes/Her dimerization and DNA binding interactions presented in this recent work does not consider any dynamics [26] and thus does not model temporal behavior of the segmentation clock. In summary, Hes/Her dimers with low DNA binding activity have not been considered in previous dynamic models of the segmentation clock, but in our new model they have important functions in determining the dynamics of the circuit, as we will explain below.

### A Protein-Protein Interaction Network Determines Clock Dynamics

Our finding that Her1 homodimers and Her7:Hes6 heterodimers target the same DNA-sites in the regulatory regions of *her1* and *her7* and within the promoter of the cyclic *dlc* gene thought to mediate coupling between single cell oscillators [10],[11],[13],[29] appears at first difficult to reconcile with the distinct phenotypes of *her1*, *her7*, and *hes6* single mutants. It is of course possible that Her1 homodimers and Her7:Hes6 heterodimers recruit distinct accessory machineries to regulate transcription at cyclic gene promoters. Alternatively, Her1, Her7, or Hes6 might gain distinct DNA binding activity by selective dimerization with bHLH factors not investigated here, and either of these scenarios might contribute to the observed phenotypic differences. However, our analysis of a mathematical model that is solely based on the experimentally determined interactions between Her1, Her7, and Hes6 suggests that this small network alone is sufficient to account for the distinct mutant phenotypes. Our finding that Hes/Her proteins dimerize promiscuously and form a “dimer cloud” that contains complexes with strong and weak DNA binding activity provides the key ingredient required to generate these phenotypic differences.

In our model, period slowing upon loss of *hes6* is caused by the influence of Hes6 on effective protein stability. Although we assume that degradation acts equally on Hes/Her monomers and dimers, the sequestration of monomers into dimers and degradation of these dimers introduces an effective monomer degradation rate that is a function of dimer concentrations, which are nonlinear functions (products) of the monomer concentrations. Experimental support for this feature of our model comes from the expression patterns of Hes6 protein. These show evidence for Hes6 protein oscillations, despite non-oscillatory *hes6* mRNA expression. Our model suggests how these protein patterns might arise—the cyclically expressed proteins Her1 and Her7 dimerize with the constantly expressed Hes6 protein and thereby cyclically modulate its effective stability. Conversely, in our model dimerization of Hes6 with Her1 and Her7 results in a shorter effective half-life of the two cyclic proteins. Consequently, in the *hes6* mutant situation, the effective stability of the oscillating proteins Her1 and Her7 is increased, and this increases the period. Previous theoretical work also shows that increased stability leads to longer oscillation period in Hes/Her feedback systems [10],[39],[40]. It will be interesting to directly investigate this hypothesis arising from our model by measuring Her1 and Her7 half-lives in wildtype and *hes6* mutant embryos. Furthermore, according to our model, Hes6 overexpression should further reduce the effective stability of Her1 and Her7 and might therefore reduce the period of the clock. This will have to be tested in transgenic fish carrying additional copies of the *hes6* gene in order to allow for controlled Hes6 overexpression and to circumvent gastrulation defects induced by *hes6* mRNA injection [18].

Dimerization-induced changes in stability are known for several bHLH proteins, where complex formation usually results in an increased half-life [41],[42]. This effect is termed cooperative stability, and theoretical studies have established that this phenomenon can affect the dynamic behavior of genetic networks [43]–[45]. In the context of the segmentation clock, it has been suggested that cooperative stability could increase the region in parameter space where sustained oscillations are possible, rendering the oscillator more robust [46]. Although different monomer and dimer stabilities can be accounted for in our model, they are not necessary to recapitulate the observed embryonic phenotypes, and we do not explore their effects here.

While the effects of Hes6 on oscillation period in our model arise from its equally destabilizing effect on both Her1 and Her7, the fact that Hes6 forms heterodimers with strong DNA binding activity only with Her7 provides an explanation for posterior segmentation defects in *her7* mutants. In the absence of Her7, more Hes6 engages in Her1:Hes6 heterodimers with low DNA binding activity, thereby reducing the pool of Her1 homodimers available for transcriptional regulation. In our model, this results in a decrease in the amplitude of oscillations, which is a plausible explanation for the posterior segmentation defects observed in *her7* mutants. To directly test this idea experimentally, it will be necessary to follow oscillations in *her7* mutants with single cell resolution.

The inactivating function of Hes6 towards Her1 in our model of the zebrafish segmentation clock contrasts with a previous report, where Hes6 was shown to increase the repressive activity of Her1 towards the *her1* promoter [18], although the duration (48 h) and biological host (293T cells) used in this experiment makes direct comparison to the segmentation clock difficult. In the developing mouse nervous system, Hes6 has been shown to inactivate the Her1 homolog Hes1 [47], analogous to its function in our model. Negative regulation of bHLH factor activity by dimerization is a well-established concept: Id factors, which lack a basic domain, can inhibit DNA binding of tissue specific bHLH factors by forming inactive heterodimers [48], and Hairy-related transcription factors can inhibit the function of lineage-specific bHLH proteins by heterodimerization [49]. However, while these well-known examples of negative regulation by dimerization occur between proteins that belong to different classes of the bHLH family, the inactivation of Hes1 by Hes6 in the mouse nervous system and the formation of dimers with lowered DNA binding activity described in this work occur between proteins of the same subclass. Furthermore, our results suggest that Hes6 has opposite effects on the closely related proteins Her7 and Her1, being a necessary dimerization partner for Her7 to gain DNA binding activity, while inhibiting DNA binding of Her1. This is a new observation for bHLH proteins, and it will be interesting to investigate which structural features determine Hes6's mode of action towards different Hes/Her factors.

The role of Hes/Her dimerization in the zebrafish segmentation clock may be relevant for understanding the mouse segmentation clock. While the overall topology of the mouse and zebrafish segmentation clock networks, including intercellular signaling pathways, has clear differences, the presence of multiple oscillating Hes/Her genes is conserved [5]. The *Hes1*, *Hes5*, and *Hes7* genes oscillate in mouse PSM, and *Hes7* is required for oscillations via a transcriptional auto-repression negative feedback loop [50]. *Hes7* mutant embryos have profoundly disrupted segmentation [51], but in contrast, *Hes1* and *Hes5* single and double mutants segment overtly normally [52]. This suggests that the Hes-based negative feedback loop topology differs between mouse and zebrafish. Based on our observations, we would expect that the Hes1 and Hes5 proteins, as well as non-oscillating bHLH proteins with ubiquitous expression [53], could participate in a dimer sequestration mechanism with Hes7 similar to the zebrafish clock, thereby potentially regulating the dynamics. Although there is currently no evidence that Hes genes regulate the period in mouse, the development of techniques to measure the dynamics of mouse segmentation with sufficient precision and temporal resolution may allow such effects to be detected.

Because of the general role of Hes6 in controlling protein stability and its differential effects on the DNA binding properties of Her1 and Her7, the activities of these proteins need to be appropriately balanced to ensure the reliable function of the zebrafish segmentation clock in the wildtype. This need to balance competing activities appears to be a common theme in different dynamic biological systems. In the developing *Drosophila* eye, the activity of a network of bHLH factors has recently been shown to direct the timing and spacing of cellular differentiation [54]. In the genetic network of the mouse circadian clock, loss of *mPer2* disrupts circadian rhythmicity, and this phenotype can be rescued by disruption of *mCry2* in *mPer2* mutant mice [55]. This rescue phenotype is similar to the restoration of segmentation clock oscillations by mutating *hes6* in a *her7* mutant background that we describe here, and by joint morpholino knockdown described recently elsewhere [26]. This suggests that, similar to control of Hes6 levels by Her7 in the segmentation clock, mPer2 levels in the circadian clock need to be held in check by mCry2.

### Integration of Multiple *her* Genes' Functions and Spatial Aspects of the Zebrafish Segmentation Clock

In this work, we take a reductionist approach to understand the identity and function of the core oscillator of the zebrafish segmentation clock: We investigate regulatory interactions between only three genes and try to understand their system-level loss-of-function phenotypes by modeling the dynamics of a small single-cell network. Our success in modeling a range of phenotypes with this small set of components, as well as the finding that joint loss of *her1* and *her7* or *her1* and *hes6* function completely abrogates oscillatory cyclic gene expression and segmentation (Figure S6) [11],[12],[31], indicates that the two-loop system described here forms the core of the zebrafish segmentation clock. Nevertheless, the dynamics of this core network may be influenced by its interaction with factors not considered here. For example, several other *hes/her* genes as well as *tbx16* display transcriptional oscillations in the PSM [5],[56],[57]. Specifically, it has been shown that the gene products of the oscillating genes *her12* and *her15* bind targets sites from the *her7* promoter in vitro both as homodimers and as heterodimers with Hes6 [26]. In addition, we show here that Hes/Her proteins can interact with non-hairy bHLH proteins such as MyoD with considerable affinity, which presumably impacts on their activity. How cyclic and non-cyclic bHLH proteins integrate into the core network formed by Her1, Her7, and Hes6 and modulate its dynamics will depend on whether they form dimers with or without DNA binding activity amongst each other and with the three proteins investigated here.

Because we focus on the regulation of oscillatory dynamics at the single cell level, we have not explicitly addressed tissue-level aspects of oscillatory gene expression in the PSM. We have previously shown that coupling of single cell oscillators through Delta-Notch signaling can modulate the period of tissue-level oscillations [58],[59]. Therefore, although our single cell model allows us to fit a range of dynamic tissue-level phenotypes, it is possible that these phenotypes do not solely arise from changes in the regulatory interactions within single cells, but might also depend on the modulation of altered single cell dynamics by cell-cell communication.

Furthermore, on the tissue level the transcriptional oscillations of the segmentation clock manifest as moving stripes of gene expression, and theoretical studies have established that this spatial aspect of the clock's oscillations can be reproduced by a gradual slowing of cell-autonomous oscillations in more anterior regions of the PSM [6],[13],[59]–[65]. While our model describes the situation in the posterior PSM, this gradual slowing could be achieved by a changing configuration of the *hes/her* gene regulatory network in more anterior parts of the PSM. The integration of oscillatory *her* genes with region-specific expression in the PSM [5],[56],[57] into the core network might provide one mechanism for the position-specific control of single-cell oscillatory period.

The graded expression of *hes6* across the tailbud and PSM (Figure 8, [18]) has been suggested to directly control the slowing of oscillations [8], but the evidence here does not obviously support this scenario. Mutating *hes6* changes the period of oscillations by only 6% [17], and theoretical work shows that such a small period change is insufficient to generate the observed wildtype wave pattern [46]. Furthermore, we found that mutating *hes6* does not grossly affect the wave patterns of cyclic gene expression (Figure 5B). In contrast, we note that the wave patterns are strongly altered in *her1* mutants, although the overall pace of segmentation is not (Figures 4 and 6). This suggests that *her1* has a primary role in period control in the anterior PSM where oscillations slow down. Conversely, in the tailbud where the pace of segmentation is determined, elevated levels of *hes6* expression may allow the Her7:Hes6 heterodimer to exert a dominant control over the circuit's period. Thus it is tempting to speculate that the wildtype zebrafish segmentation clock may use changing Hes6 expression levels to switch between the two core oscillatory negative feedback loops in a position-dependent manner across the PSM.

In summary, combining biochemical and genetic data with mathematical modeling, we have developed a model for the zebrafish segmentation clock's core circuit with a novel regulatory topology and an unexpectedly prominent role for a “dimer cloud” of Hes/Her complexes in regulating the formation and availability of DNA binding dimers. Oscillating systems generally must display (i) negative feedback, (ii) delays in this feedback, (iii) sufficient nonlinearity, and (iv) a balance of timescales [66]. Our biochemical measurements of DNA-binding Her1 homodimers and Her7:Hes6 heterodimers onto target sites from oscillating gene promoters reveal the redundant two-loop topology of the negative feedback in the core circuit, as well as a likely source of strong non-linearity via the existence of multiple binding sites [67]. This redundant two-loop topology may provide the circuit with robustness to genetic and environmental perturbation, while the distinct components of each loop could provide the core circuit with independent input and/or output regulatory linkage that might vary with the position of the cell in the PSM, or with developmental stage, or through evolutionary transitions. The balance of timescales in the system is controlled in a surprising way: Hes6 acts to tune this balance by regulating the effective degradation of the oscillating DNA binding dimers, thereby changing the period of the clock. This global regulation of the stability of the oscillating components, and hence the period, through protein-protein interaction with a component whose levels can be smoothly and gradually tuned by external signals is a new control mechanism for the segmentation clock. How widely this principle is employed in other genetic regulatory systems remains to be explored.

## Materials and Methods

### Expression Constructs and MITOMI Target DNAs

*her1*, *hes6*, *her7*, and *myoD* coding sequences were PCR-amplified from wildtype zebrafish cDNA, cloned into pDON221 entry vectors using Gateway technology, and verified by sequencing. pMARE vectors for in vitro expression of C-terminally GFP-tagged proteins have been described [68]. To generate pMARE vectors for in vitro expression of mCherry- and GST-tagged bHLH proteins, the GFP-coding sequence of pMARE was replaced by the respective mCherry or GST coding sequence. *her1*, *hes6*, *her7*, and *myoD* coding sequences were subcloned into pMARE via a Gateway LR-reaction. Expression constructs for yeast one-hybrid experiments were generated by LR-subcloning of *her1*, *her7*, and *hes6* cDNAs into pDEST_AD_ [25] or pDEST_AD-DIMER_. The generation of pDEST_AD-DIMER_ will be described elsewhere (Hens et al., in preparation). Target DNA sequences for MITOMI experiments were Cy5-labeled as previously described [19].

### MITOMI Experiments

pMARE expression vectors or labeled target DNAs in a carrier solution containing 1% BSA were deposited onto epoxy coated glass sub-strates (CEL Associates) using a QArray Microarray spotter. MITOMI experiments were performed using microfluidic chips with 768 reaction chambers. Flow and control molds and microfluidic devices were fabricated, aligned, and bonded to spotted slides as previously described [19],[69], and initial surface derivatization was performed according to published protocols [19]. Antibodies to immobilize tagged Her-proteins on the chip surface were anti-GST and anti-GFP (abcam). For protein-protein interaction experiments, 12.5 µL of a TnT SP6 high-yield wheat germ protein expression extract (Promega) were programmed with 500 ng of expression vector encoding C-terminally GFP-tagged Her-proteins, incubated for 3 h to express protein and loaded onto the chips with buttons closed. Flow was then stopped and buttons opened to allow pulldown of GFP-tagged Her protein to the button area from the volume corresponding to one unit cell. Excess GFP-tagged protein was removed by extensive washing with PBS. Next, unprogrammed wheat germ extract containing 250 nM of a Cy5-labeled DNA-oligomer containing the CGACACGTGCTC sequence was loaded onto the device and flushed for 5 min, after which the chamber valves were opened allowing for dead end filling of the chambers with the expression extract [20]. For experiments testing the interaction of one dimer combination with multiple DNA-sequences, chambers were filled directly after depositing the antibody to the button surface with a wheat germ extract programmed with the respective expression constructs. Devices were incubated for 3 h at room temperature to allow for protein expression and equilibrium binding. Device imaging and image and data analysis were performed as previously described [19]. Data fitting and statistical analysis of the fits was performed in GraphPad Prism. Error bars state standard error of the linear fits.

### Yeast One-Hybrid

Eighteen fragments between approximately 100 bp and 1 kb in length upstream of the *her1*, *her7*, and *dlc* start codon were PCR-amplified from genomic zebrafish DNA, introduced into pDONR-P1P4R using Gateway technology, sequence verified, and subcloned into pDB-DEST-His and pDB-DEST-LacZ [25]. Y1H bait strains were generated and tested for self-activation as described [25]. Reporter strains were transformed with pDEST_AD_ or pDEST_AD-DIMER_ vectors encoding N-terminal Gal4-AD fusions of different Her-proteins according to standard procedures, and transformants selected by growth on media lacking tryptophan. Yeast cells were arrayed using a Singer Rotor pinning robot, and interactions determined by assaying growth on 3-aminotriazole containing minimal media or β-gal expression according to standard procedures.

### Fish Care and Genotyping

Wildtype and mutant zebrafish were maintained according to standard procedures and embryos obtained by natural spawning. *hes6* mutants have previously been described [17]. The *her1^hu2124^* and the *her7^hu2526^* alleles were generated by ENU mutagenesis [28] and distributed in the framework of the ZF-MODELS project. Carriers of the *her1* and *her7* mutant alleles were continuously backcrossed to the AB wt strain to reduce background mutations. Backcrossing improved longevity of adult fish but did not impact on selected embryonic phenotypes such as anterior or posterior segmentation defects in the *her1* or *her7* mutant, respectively. Homozygous *her7* mutants for raising and double homozygous *her1;hes6* mutants were identified visually by scoring for segmentation defects around the 18-somite stage. *her7* mutants in period measurement experiments and all other mutants were identified by PCR-based genotyping protocols. Genomic DNA was isolated from tissue samples (adult fish) or whole embryos using standard procedures [28]. *her1* mutant fish were identified by digesting a PCR amplicon covering the *hu2124* lesion with TfiI. This restriction site is present in the wildtype, but not the mutant allele. *her7;hes6* double mutants were identified by sequencing of a PCR amplicon of the *her7* locus covering the *hu2526* lesion and PCR genotyping of the *hes6* lesion as previously described [17]. Primer sequences and reaction conditions for all PCR-based genotyping protocols are available from the authors upon request. All single and double mutants described in this work were homozygous viable and fertile.

Embryos for period measurements in single mutants and myotome counts in *hes6* heterozygous mutants were obtained from incrosses of heterozygous carriers of the respective lesion. Embryos for analyzing somitogenesis period in *her7;hes6* double mutants were obtained by crossing trans-heterozygous and double homozygous carriers. Experiments addressing how the onset of segmentation defects in *her7* mutants depends on *hes6* zygosity were performed on embryos from incrosses of *her7* homozygous;*hes6* heterozygous adults. Embryos to determine somitogenesis period, myotome number, and position of segmentation defects were individually genotyped after analysis to eliminate potential analyzer bias. Embryos for all other experiments were obtained from incrossing of homozygous carries of the desired genotype.

### In-Situ Hybridization, Documentation, and Scoring of Fixed Embryos

Antisense probes to *her1* [70], *her7*, *dlc* [11], *cb1045* [71], *hes6* [17], and *myoD* [72] have been described. In situ hybridization using NBT/BCIP and FastRed chemistry was performed as described [11],[73]. For fluorescent in situ staining using tyramide signal amplification (TSA), riboprobe hybridization, washes, and antibody incubation were carried out as described above, except that peroxidase-coupled anti-digoxigenin antibody (Roche) was used. Color development was performed with Cy3-coupled tyramide according to the manufacturer's recommendations (Perkin Elmer). Cy-3 stained embryos were cleared with methanol, deyolked, and imaged on a Zeiss Axioskop 200 M equipped with a Photometrics Coolsnap HQ Camera and a motorized stage (Zeiss MCU 28) driven by MetaMorph software (version 6.2r4, Universal Imaging Corp.). Z-stacks were deconvolved with Huygens software (Scientific Volume Imaging), and maximum intensity projections generated from deconvolved stacks. All other in situ stained embryos were photographed either in whole mount on an Olympus SZX12 stereomicroscope equipped with a QImaging Micropublisher 5.0 RTV camera or flat mounted and photographed on a Zeiss Axioskop 2 equipped with a Retiga SRV camera (QImaging). Images were processed in Photoshop and ImageJ. Intensity profile plots were measured in ImageJ in two rectangular boxes on either side of the notochord that were 50 µm wide and extended 315 µm from the posterior end of the notochord. Segmentation defects and myotome number were scored in embryos stained with a *cb1045* riboprobe as described in [74] and [17], respectively.

### Period Measurements

Somitogenesis period was determined by multiple-embryo time-lapse imaging as previously described [32],[33]. Briefly, somitogenesis movies were analyzed visually by annotating the time of boundary formation for somites 2–17 in *her1* and *hes6* mutants or somites 2–10 in *her7* mutants. Somitogenesis period in minutes was calculated in Microsoft Excel for each embryo individually from the slope of the linear fit to the data points. Period measurements were subsequently normalized by dividing through the mean of the period measurements of the wildtype control population in each experiment, yielding the non-dimensional normalized somitogenesis period. The 95% confidence intervals were calculated in Microsoft Excel, and Mann-Whitney test was used to assess significance.

### Modeling

Our mathematical model is based on delay differential equations describing the dynamics of Her1, Hes6, and Her7 proteins and all their possible dimers. Using an equilibrium approximation that assumes that Hes/Her dimerization is faster than the other processes in the system [22], we have reduced this model to only three equations describing the change of concentrations with time *s*, of monomers *h*_1_, *h*_6_, and *h*_7_:
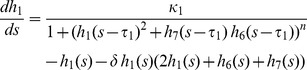


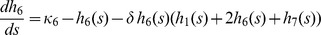


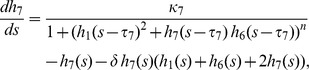
where the production delays associated with synthesis of Her1 and Her7 are *τ_1_* and *τ_7_*, respectively; the production rates of Her1, Her7, and Hes6 are *κ_1_*, *κ_2_*, and *κ_6_*, respectively; *n* is a phenomenological Hill coefficient describing effective cooperativity arising from multiple binding sites for Hes/Her dimers in each of the promoters; and *δ* is an effective dimer-mediated degradation rate. Details of the derivation, parameterization, and simulation of the model are given in the Text S1.

### Generation of Monoclonal Antibodies and Immunohistochemistry

Recombinant full-length Hes6 protein was expressed in bacteria, purified, and used to immunize mice according to standard procedures. Complete antisera were screened for Hes6 binding, and one mouse was selected for boosting and production of hybridoma cell lines. For our experiments we used a mixture of the purified supernatants from two hybridoma cell lines that both gave specific immunostaining in the PSM of 10-somite stage embryos. Immunostaining was performed according to standard procedures. Briefly, embryos were fixed for 2 h in 2% paraformaldehyde, dechorionated, and permeabilized for several hours in methanol. Following rehydration, embryos were blocked in 10% BSA/1% DMSO for 4 h and incubated with primary antibodies at a concentration of 0.8 µg/ml at 4°C for several hours. Secondary antibodies were peroxidase-coupled anti-mouse IgG at 1∶1,000 dilution. Color development was performed with Cy3-coupled tyramide according to the manufacturer's recommendations (Perkin Elmer). Embryos were cleared with methanol, flat mounted, and imaged on a Zeiss Axioskop 2 equipped with a Retiga SRV camera (QImaging) or on a Zeiss LSM510 confocal system. Images were processed in Photoshop and ImageJ.

## Supporting Information

Figure S1 Promiscuous interaction between Her1, Her7, and Hes6. GFP-tagged Her1 (left), Her7 (middle), or Hes6 (right) was co-expressed with different mCherry-tagged proteins in an in vitro transcription-translation system as indicated. GFP-tagged proteins were purified by immunoprecipitation using a GFP-antibody and transferred to membranes. Probing for mCherry-tagged proteins (upper panels) reveals that all proteins containing a basic helix-loop-helix domain (i.e., MyoD, Her1, Her7, and Hes6) are co-purified with the GFP-tagged protein significantly stronger than the negative control PPARγ but that there is little difference in co-purification efficiency between different bHLH proteins. This indicates that the bHLH proteins investigated here interact promiscuously. IP, immunoprecipitation; IB, immunoblotting.
(TIF)

Figure S2 Co-expression of Her7-mCherry or Hes6-mCherry does not alter the binding energy landscape of Her1-GFP. Relative binding affinities of Her1-GFP towards 47 different NNNCACGNGNNN sites from cyclic gene promoters were determined by MITOMI, and the value of the strongest binder in the library was normalized to one. Each data point represents one sequence, and the relative affinity towards Her1-GFP in the presence of PPARγ-mCherry is plotted against the relative affinity of that site towards Her1-GFP in the presence of Her7-mCherry (A) or Hes6-mCherry (B). Data points cluster around the line representing equal affinities (dashed red line), suggesting that presence of Her7 or Hes6 does not alter the binding energy landscape of Her1.
(TIF)

Figure S3 Tissue-level transcriptional oscillations in *hes6 *mutant embryos. Wildtype (wt, upper row) and *hes6* mutant (lower row) embryos at the 10-somite stage in situ stained for *her7* (A), *dlc* (B), or *her1* (C) mRNA expression (blue). in situ staining for *myoD* expression (red) marks formed somites. Flat mount preparations, anterior to the top, scale bar 100 µm. Alternating patterns indicative of tissue-level oscillatory gene expression are evident for each probe. This is in contrast to a previous study, where MO-mediated *hes6* knockdown resulted in loss of oscillatory expression of *her1*, *her7*, and *dlc* [18]. These discrepancies could be caused by off-target effects of the MOs used in [18] or by raising the embryos at different temperatures in the two studies. Note that the embryos shown here were raised at 28.5°C, where the majority of *hes6* mutant embryos segments normally [17].
(TIF)

Figure S4 The *her1^hu2124^* and the *her7^hu2625^* alleles lead to full loss of *her1* and *her7* function, respectively. (A) Schematic representation of the genomic organization of the *her1* locus. Boxes represent exons, and lines represent introns (distances not to scale). An asterisk indicates the approximate position of a nonsense mutation in the *hu2124* allele that was generated by ENU mutagenesis [28] at the Hubrecht laboratory (Netherlands). Carriers of the *her1^hu2124^* allele are referred to as *her1* mutant in this work and were homozygous viable and fertile. The mutant stop codon disrupts the bHLH domain, which is encoded within the first three exons. (B) Sequencing trace from heterozygous carriers of the *hu2124* allele. The C-to-T exchange is evident, changing the codon from Ser to stop. (C) To study whether *her1^hu2124^* lead to full loss of *her1* function, wildtype (wt) and *her1* mutants were injected with a combination of *her1* targeted morpholino antisense oligonucleotides (MOs) or left uninjected, grown to 34 hpf, and stained with the myotome boundary marker *cb1045*. *her1*MO injection into wt and the *her1* mutant results in partially penetrant anterior segmentation defects similar to the uninjected *her1* mutant. Scale bars, 300 µm (big panels) and 50 µm (insets). (D) The percentage of defective posterior boundaries for each segment along the anterior trunk was determined in groups of embryos treated as in (C). Combining the mutant allele and MO-mediated knockdown does not increase the penetrance or severity of segmentation defects, suggesting that *her1* function is fully lost in all three conditions. Data are pooled from two (wt) or three (*her1* mutant) independent experiments. (E) Schematic representation of the genomic organization of the *her7* locus. Boxes represent exons, and lines represent introns (distances not to scale). An asterisk indicates the approximate position of the nonsense mutation in the *hu2625* allele that was generated by ENU mutagenesis [28] at the Hubrecht laboratory (Netherlands). Carriers of the *her7^hu2625^* allele are referred to as *her7* mutant in this work, and homozygous carriers were viable and fertile. The premature stop codon in *her7* mutants is located within the HLH domain that mediates dimerization between bHLH proteins. (F) Sequencing trace from heterozygous carriers of the *hu2625* allele. The A-to-T exchange is evident, changing the 38^th^ codon of the Her7 protein from Lys to stop. (G) wt and *her7* mutant embryos were injected with *her7* targeted MOs or left uninjected, grown to 34 hpf, and stained with the myotome boundary marker *cb1045*. Segmentation defects posterior to a similar axial level (arrowhead) are evident in *her7*MO injected wt embryos as well as uninjected and injected *her7* mutant embryos. Scale bar 300 µm. (H) The Anterior Limit of Defects (ALD) [74],[75] was scored in groups of *her7*MO injected wt embryos and injected or uninjected *her7* mutant embryos to exactly quantify the severity of the segmentation defects. Combination of MO-mediated knockdown and the *her7* mutant allele shifts the ALD anteriorly by less than one segment. Although it cannot be excluded that this slight shift is due to the knockdown of some residual *her7* activity in the mutant, these data suggest that *her7* function is almost completely absent in homozygous carriers of the *her7^hu2526^* allele. Data were pooled from two independent experiments; error bars indicate standard deviation.
(TIF)

Figure S5 Tissue-level transcriptional oscillations in *her1* and *her7* mutant embryos at the bud stage. (A, B) Wildtype (wt, upper row) and *her1* mutant (lower row) embryos at the bud stage in situ stained for *her7* (A) or *dlc* (B) mRNA expression. (C–E) wt (upper row) and *her7* mutant (lower row) embryos at the bud stage in situ stained for *her7* (C), *dlc* (D), or *her1* (E) mRNA expression. Two representative examples per condition shown. Alternating patterns indicative of tissue-level oscillatory gene expression are evident for each genotype and probe. Whole mount preparations, anterior to the top, scale bars, 100 µm.
(TIF)

Figure S6 Combined loss of *her1* and *hes6* or *her1* and *her7* function fully disrupts segmentation and tissue-level oscillatory *her7* expression. (A) Wildtype (wt), *her1* mutant, *hes6* mutant, and *her1;hes6* double mutant embryos grown to 34 hpf and stained with the myotome boundary marker *cb1045* to analyze segmentation. wt and the majority of *her1* and *hes6* single mutant embryos segment normally along the entire axis, whereas all *her1;hes6* double mutant embryos display segmentation failure along the entire axis. Scale bar, 300 µm. (B) wt, *her1* mutant, *hes6* mutant, and *her1;hes6* double mutant embryos at the 10-somite stained for *her7* mRNA expression. Alternating wave patterns indicative for tissue-level oscillatory expression can be observed for wildtype, *her1*, and *hes6* single mutant embryos (two representative examples shown for each genotype), but 26 out of 27 *her1;hes6* double mutants display an equal level of *her7* expression throughout the PSM. Scale bar, 100 µm. (C) wt and *her7* mutant embryos were injected with *her1*-targeted MOs or left uninjected, grown until 34 hpf, and stained with the myotome boundary marker *cb1045* to analyze segmentation phenotypes. All wildtype and the majority of *her1* morphant embryos segment normally in the central trunk and tail, whereas *her7* mutants display posterior segmentation defects. These defects are enhanced by injection of *her1*-targeted MOs into the mutant background, which leads to segmentation failure along the entire axis. Scale bar, 300 µm. (D) Uninjected and *her1*-MO-injected wt and *her7* mutant embryos at bud-stage stained for *her7* mRNA expression. Alternating wave patterns indicative for tissue-level oscillatory expression can be observed for uninjected and *her1*-MO-injected wt and uninjected *her7* mutant embryos (two representative examples per condition shown), but *her1* MO injection into *her7* mutants leads to even *her7* expression throughout the PSM (40 out of 40, one representative example shown). Scale bar, 100 µm.
(TIF)

Figure S7 The difference between the production delays of Her1 and Her7 tunes the difference between wildtype and *her7* loss-of-function periods. Amplitude (first row) and period (second row) of the oscillations of total Her1 protein concentration *h*_1_, as a function of the dimensionless production rate of Hes6, *κ*_6_, for three different values of the dimensionless production delay of Her1: *τ*_1_ = 1∶00 (first column), *τ*_1_ = 1∶02 (second column), and *τ*_1_ = 1∶04 (third column). The amplitude is defined as the maximum minus the minimum of *h*_1_ at steady state. All the other parameters as given in Table S3 for the blue line; same for the green line except *κ*_7_ = 0.
(PDF)

Figure S8 Sensitivity analysis shows that the model is robust to changes in parameters around the values chosen to describe the wildtype condition. See accompanying Text S1 for details.
(PDF)

Figure S9 The dimensionless production rate of Hes6 can change the effective degradation rate of monomers. Average effective degradation rate (top) and average effective half-life (bottom) of Her1 monomer, as defined in Eq. (24). All the other parameters as given in Table S3 for the blue line, and same for the green line except *κ*_7_ = 0.
(PDF)

Figure S10 Average levels of Hes/Her monomers and dimers in wildtype and mutant conditions. (A) Comparison between the wildtype (grey bars) and the *her7* mutant condition (green bars). Levels of all monomers and dimers are shown. (B) Levels of Her1 homodimer (red bars) and Her7:Hes6 heterodimer (cyan bars) in the different mutant conditions. The blue line shows the level at which negative feedback halves the production rate of Her1 and Her7.
(PDF)

Figure S11 *her1* loss of function has a similar period to wildtype. Amplitude (top) and period (bottom) of the oscillations of total Her7 protein concentration *h*_7_, as a function of the dimensionless production rate of Hes6, *κ*_6_. The amplitude is defined as the maximum minus the minimum of *h*_7_ at steady state. All the other parameters as given in Table S3 for the blue line, and same for the green line except *κ*_1_ = 0.
(PDF)

Figure S12 Quantitative effects of *hes6* dosage on clock function. (A) Somitogenesis period measured by time-lapse imaging of embryos obtained from incrosses of heterozygous *hes6* mutants. Period of wildtype (wt) embryos were normalized to one, and period of homozygous *hes6* mutants is from [17], for comparison. Data pooled from three independent experiments, *n*≥19 for each genotype. Heterozygous *hes6* mutants segment 2% slower than their wt siblings, while homozygous *hes6* mutants segment 6% slower. (B) wt, heterozygous, and homozygous *hes6* mutants at 48 hpf stained for *cb1045* expression to count myotome number. The 10^th^, 20^th^, and last myotome is indicated for each genotype. (C) Quantification of myotome number in embryos stained as in (B) from incrosses of heterozygous *hes6* mutants. Myotome number was scored by an observer blind to the embryos' genotype. Heterozgygous *hes6* mutants have fewer segments than their wt siblings. Data pooled from two independent experiments, *n*≥26 per gentoype. (D) *her7* homozygous mutants with a wt or heterozygous mutant *hes6* locus at 34 hpf stained for *cb1045* expression to determine anterior limit of segmentation defects (ALD, red arrow). (E) Quantification of ALD in embryos from an incross of *her7* homozygous;*hes6* heterozygous mutants. ALDs were scored by an observer blind to the embryos' genotype. The onset of segmentation defects in *hes6* heterozygous *her7* mutants is shifted toward the posterior compared to *her7* mutants with two wildtype *hes6* alleles. Data shown are from one representative experiment, *n*≥15 per genotype. ** and * indicate *p*≤0.01 and *p*≤0.05, respectively, as determined by two-tailed Mann-Whitney *U*-test. Error bars indicate 95% confidence interval. Scale bars 300 µm.
(TIF)

Table S1 Relative binding affinities of NNNCACGNGNNN sequences to Her1 and relation between MITOMI and Y1H results. The column “12-mer” lists the sequences corresponding to sequence numbers given in Figure 2C in the main text. The central 6-mer consensus sequence for each 12-mer is given in the column “Core.” Sequences 43 to 47 are negative controls that lack a central CACGNG consensus site. Values in the columns “binding to Her1” are from a MITOMI experiment where Her1-GFP was coupled to the chip in the presence of PPARγ-mCherry and were calculated by plotting free versus bound DNA for each sequence. The column “rel. affinity” gives the slopes of the linear fits to the data points for each sequence, with the slope for the highest affinity binder (sequence 2, TGGCACGTGTCC from *dlc* promoter) normalized to 1.0. Adjacent columns state the normalized standard error and the *R* ^2^ values of the linear fits. This dataset was used to generate the bar chart in Figure 2C of the main text. The columns “binding to Her7:Hes6” give the respective values corresponding to the experiment underlying Figure 2D. The position of each sequence in the respective promoters (“distance to ATG”) is given relative to the gene's start codon, and where applicable, the Y1H bait fragment containing the 12-mer sequence is stated. Sequence 24 occurs twice in the *her7* promoter and was contained in two independent Y1H baits. Where applicable, interaction of fragments with Her1 in Y1H is stated as determined by the data given in Figure 3A–C in the main text. Y, yes; N, no; n.a., not applicable.
(XLSX)

Table S2 Parameters of the full model, Eqs. (1–9).
(PDF)

Table S3 Parameters of the minimal model in Eqs. (14–16).
(PDF)

Text S1 Minimal asymmetric model of the gene regulatory network of the zebrafish segmentation clock. *Includes* Figures S7, S8, S9, S10, S11; Table S2: Parameters of the full model, Eqs. (1–9); and Table S3: Parameters of the minimal model, Eqs. (14–16).
(PDF)

## Abbreviations

AD: activation domain
bHLH: basic helix loop helix
dlc: deltaC
ENU: N-ethyl-N-nitrosourea
GFP: green fluorescent protein
FGF: fibroblast growth factor
Hes/Her: Hairy and Enhancer of split related
hpf: hours post fertilization
mCry2: mouse Cryptochrome 2
MITOMI: mechanically induced trapping of molecular interactions
MO: morpholino oligonucleotide
mPer2: mouse Period 2
MyoD: myogenic differentiation 1
PPARγ: peroxisome proliferator-activated receptor gamma
PSM: presomitic mesoderm
tbx16: T-box 16
wt: wildtype
Y1H: yeast one-hybrid
