# Supplementary figures and images for: Topology and Dynamics of the Zebrafish Segmentation Clock Core Circuit
Source: PLoS Biol. 2012 Jul 24;10(7):e1001364. doi: 10.1371/journal.pbio.1001364 (PMC3404119; doi:10.1371/journal.pbio.1001364)

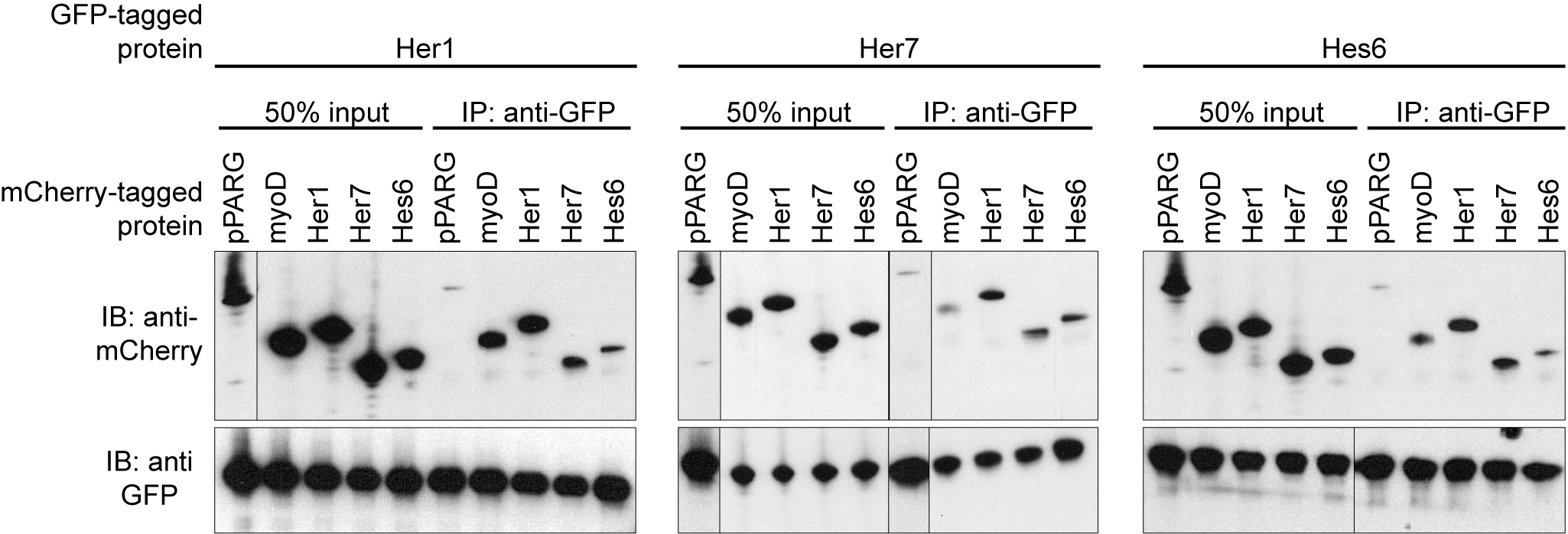

Supplement: Figure S1 — Promiscuous interaction between Her1, Her7, and Hes6. GFP-tagged Her1 (left), Her7 (middle), or Hes6 (right) was co-expressed with different mCherry-tagged proteins in an in vitro transcription-translation system as indicated. GFP-tagged proteins were purified by immunoprecipitation using a GFP-antibody and transferred to membranes. Probing for mCherry-tagged proteins (upper panels) reveals that all proteins containing a basic helix-loop-helix domain (i.e., MyoD, Her1, Her7, and Hes6) are co-purified with the GFP-tagged protein significantly stronger than the negative control PPARγ but that there is little difference in co-purification efficiency between different bHLH proteins. This indicates that the bHLH proteins investigated here interact promiscuously. IP, immunoprecipitation; IB, immunoblotting. (TIF) [file pbio.1001364.s001.tif]

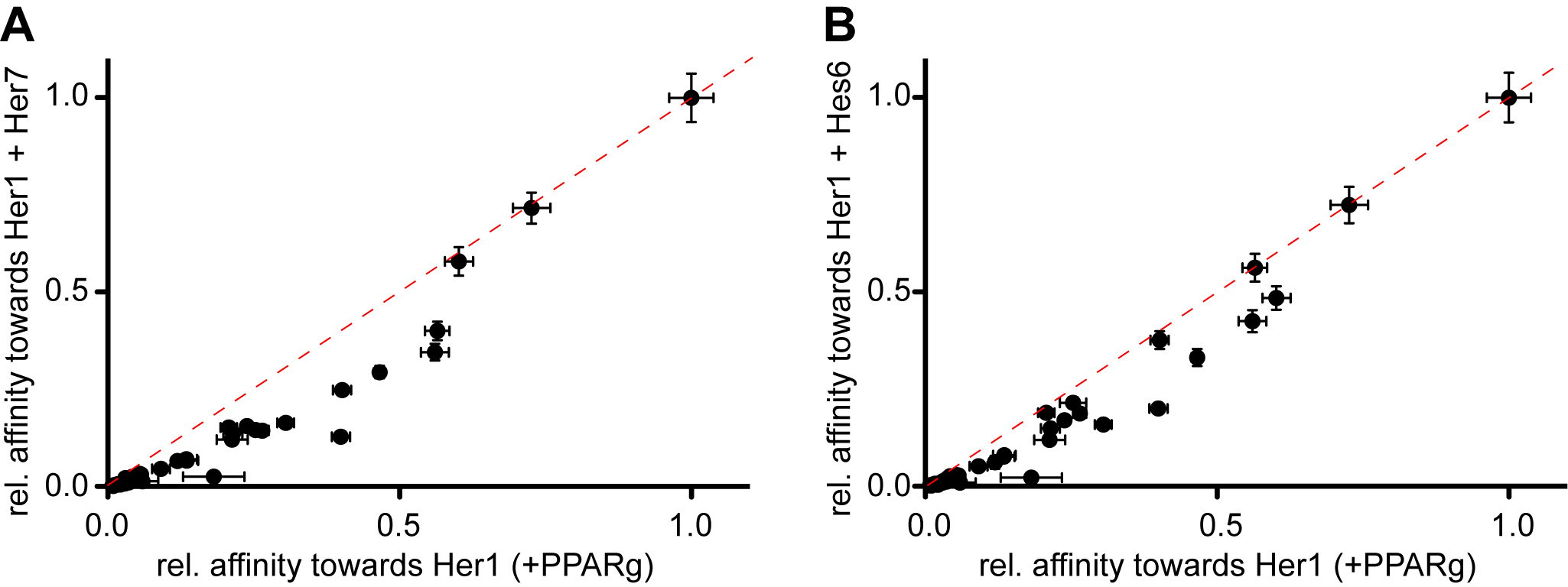

Supplement: Figure S2 — Co-expression of Her7-mCherry or Hes6-mCherry does not alter the binding energy landscape of Her1-GFP. Relative binding affinities of Her1-GFP towards 47 different NNNCACGNGNNN sites from cyclic gene promoters were determined by MITOMI, and the value of the strongest binder in the library was normalized to one. Each data point represents one sequence, and the relative affinity towards Her1-GFP in the presence of PPARγ-mCherry is plotted against the relative affinity of that site towards Her1-GFP in the presence of Her7-mCherry (A) or Hes6-mCherry (B). Data points cluster around the line representing equal affinities (dashed red line), suggesting that presence of Her7 or Hes6 does not alter the binding energy landscape of Her1. (TIF) [file pbio.1001364.s002.tif]

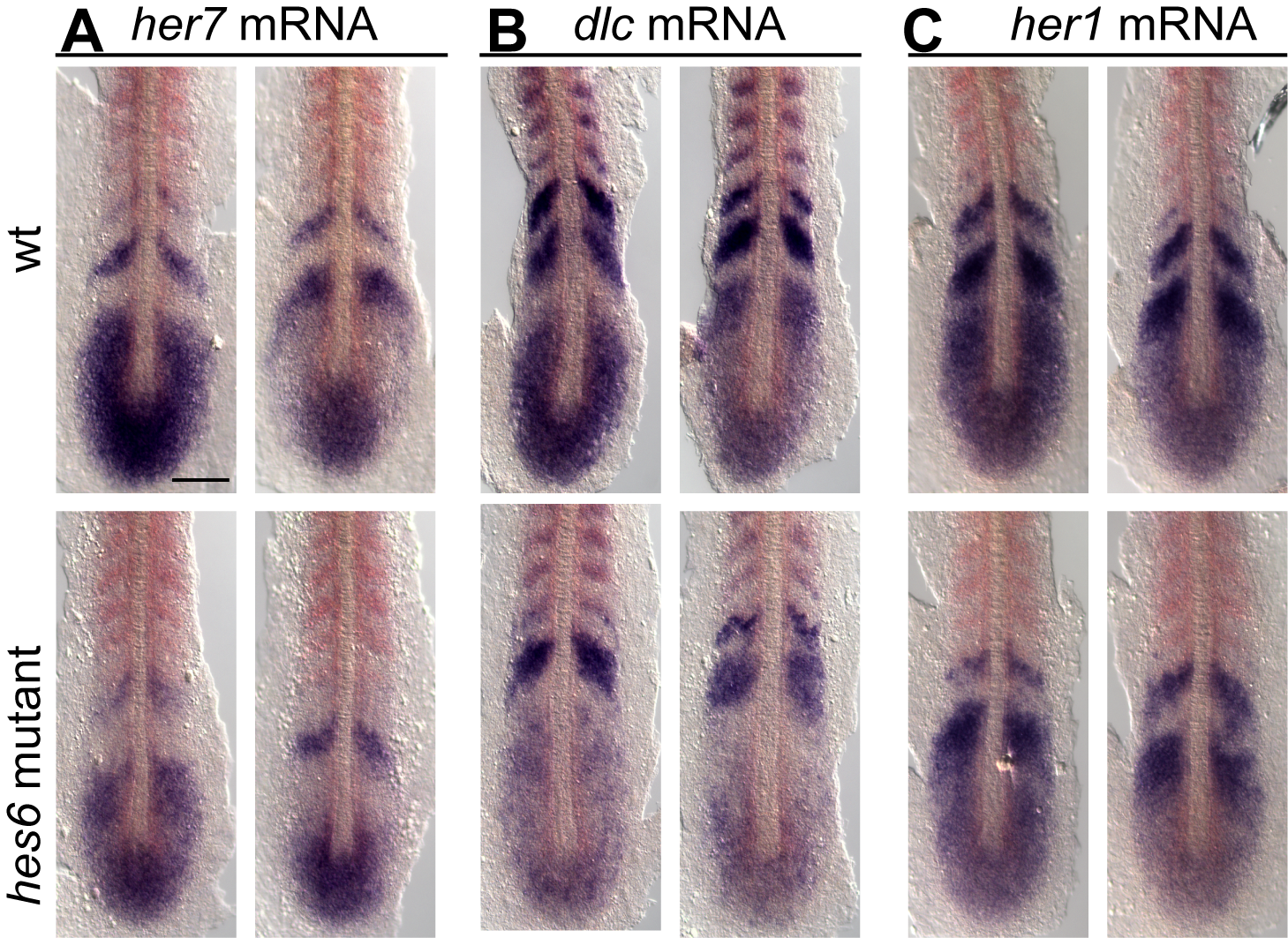

Supplement: Figure S3 — Tissue-level transcriptional oscillations in hes6 mutant embryos. Wildtype (wt, upper row) and hes6 mutant (lower row) embryos at the 10-somite stage in situ stained for her7 (A), dlc (B), or her1 (C) mRNA expression (blue). in situ staining for myoD expression (red) marks formed somites. Flat mount preparations, anterior to the top, scale bar 100 µm. Alternating patterns indicative of tissue-level oscillatory gene expression are evident for each probe. This is in contrast to a previous study, where MO-mediated hes6 knockdown resulted in loss of oscillatory expression of her1, her7, and dlc [18]. These discrepancies could be caused by off-target effects of the MOs used in [18] or by raising the embryos at different temperatures in the two studies. Note that the embryos shown here were raised at 28.5°C, where the majority of hes6 mutant embryos segments normally [17]. (TIF) [file pbio.1001364.s003.tif]

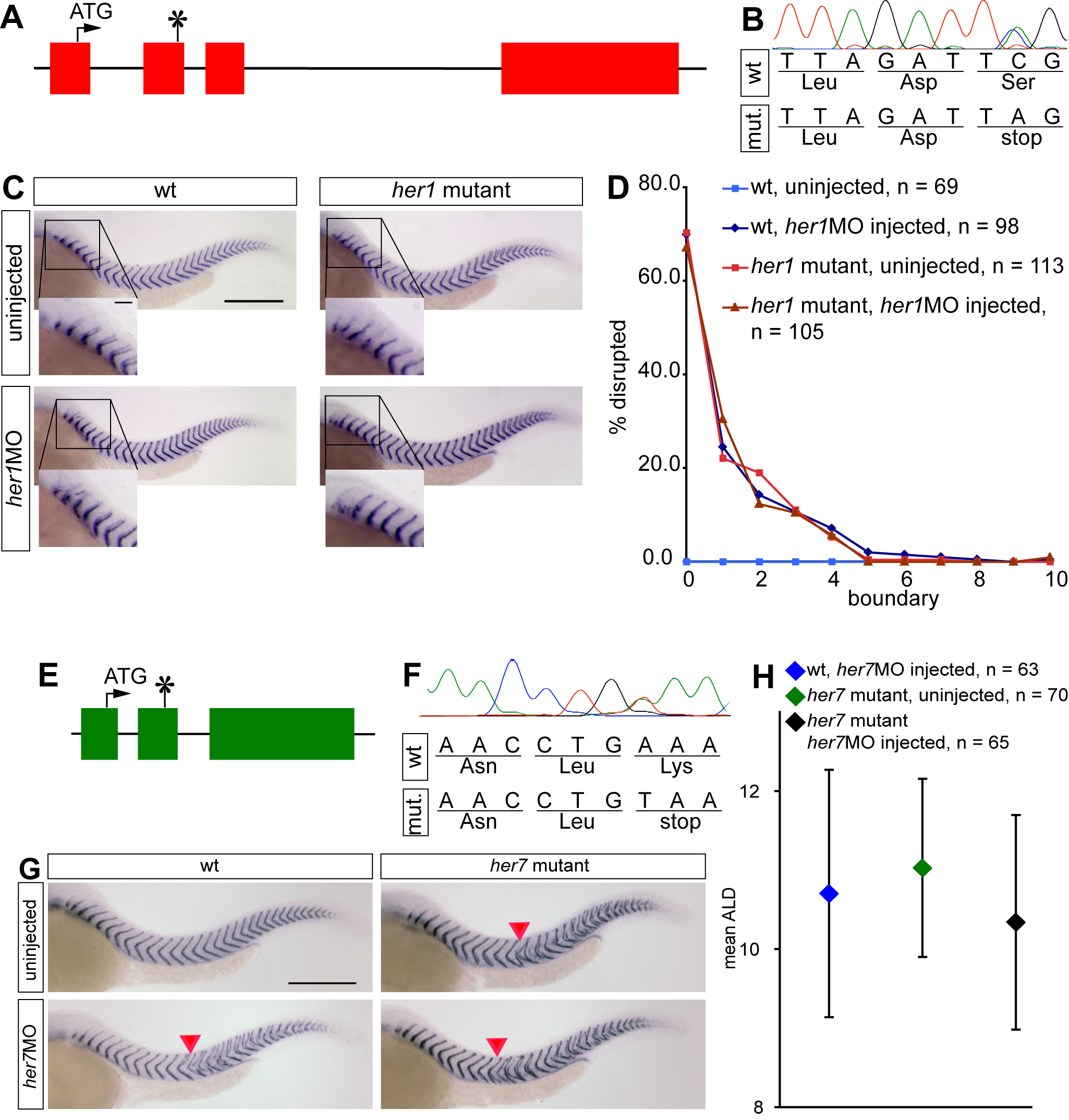

Supplement: Figure S4 — The her1hu2124 and the her7hu2625 alleles lead to full loss of her1 and her7 function, respectively. (A) Schematic representation of the genomic organization of the her1 locus. Boxes represent exons, and lines represent introns (distances not to scale). An asterisk indicates the approximate position of a nonsense mutation in the hu2124 allele that was generated by ENU mutagenesis [28] at the Hubrecht laboratory (Netherlands). Carriers of the her1hu2124 allele are referred to as her1 mutant in this work and were homozygous viable and fertile. The mutant stop codon disrupts the bHLH domain, which is encoded within the first three exons. (B) Sequencing trace from heterozygous carriers of the hu2124 allele. The C-to-T exchange is evident, changing the codon from Ser to stop. (C) To study whether her1hu2124 lead to full loss of her1 function, wildtype (wt) and her1 mutants were injected with a combination of her1 targeted morpholino antisense oligonucleotides (MOs) or left uninjected, grown to 34 hpf, and stained with the myotome boundary marker cb1045. her1MO injection into wt and the her1 mutant results in partially penetrant anterior segmentation defects similar to the uninjected her1 mutant. Scale bars, 300 µm (big panels) and 50 µm (insets). (D) The percentage of defective posterior boundaries for each segment along the anterior trunk was determined in groups of embryos treated as in (C). Combining the mutant allele and MO-mediated knockdown does not increase the penetrance or severity of segmentation defects, suggesting that her1 function is fully lost in all three conditions. Data are pooled from two (wt) or three (her1 mutant) independent experiments. (E) Schematic representation of the genomic organization of the her7 locus. Boxes represent exons, and lines represent introns (distances not to scale). An asterisk indicates the approximate position of the nonsense mutation in the hu2625 allele that was generated by ENU mutagenesis [28] at the Hubrecht laboratory ( [file pbio.1001364.s004.tif]

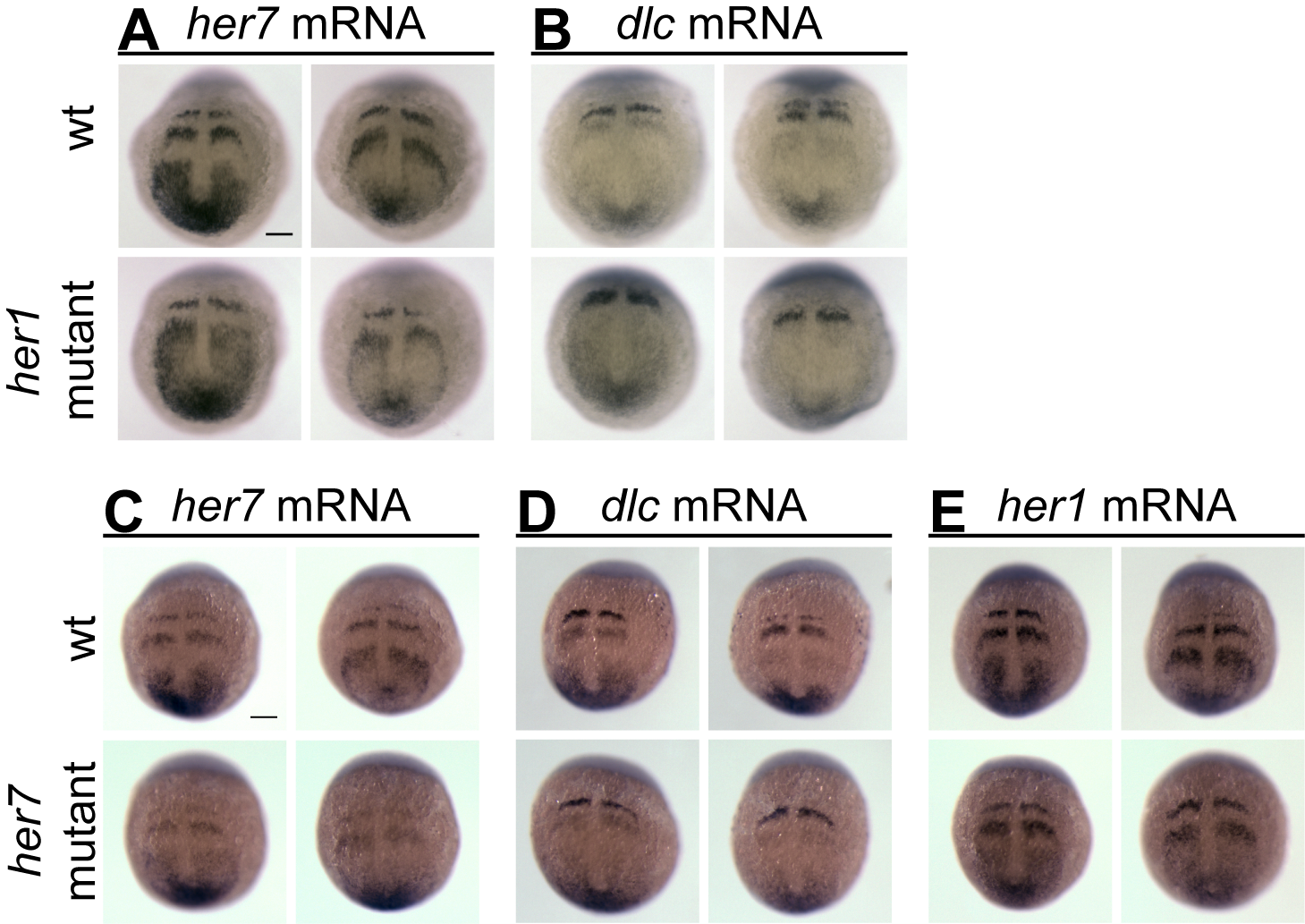

Supplement: Figure S5 — Tissue-level transcriptional oscillations in her1 and her7 mutant embryos at the bud stage. (A, B) Wildtype (wt, upper row) and her1 mutant (lower row) embryos at the bud stage in situ stained for her7 (A) or dlc (B) mRNA expression. (C–E) wt (upper row) and her7 mutant (lower row) embryos at the bud stage in situ stained for her7 (C), dlc (D), or her1 (E) mRNA expression. Two representative examples per condition shown. Alternating patterns indicative of tissue-level oscillatory gene expression are evident for each genotype and probe. Whole mount preparations, anterior to the top, scale bars, 100 µm. (TIF) [file pbio.1001364.s005.tif]

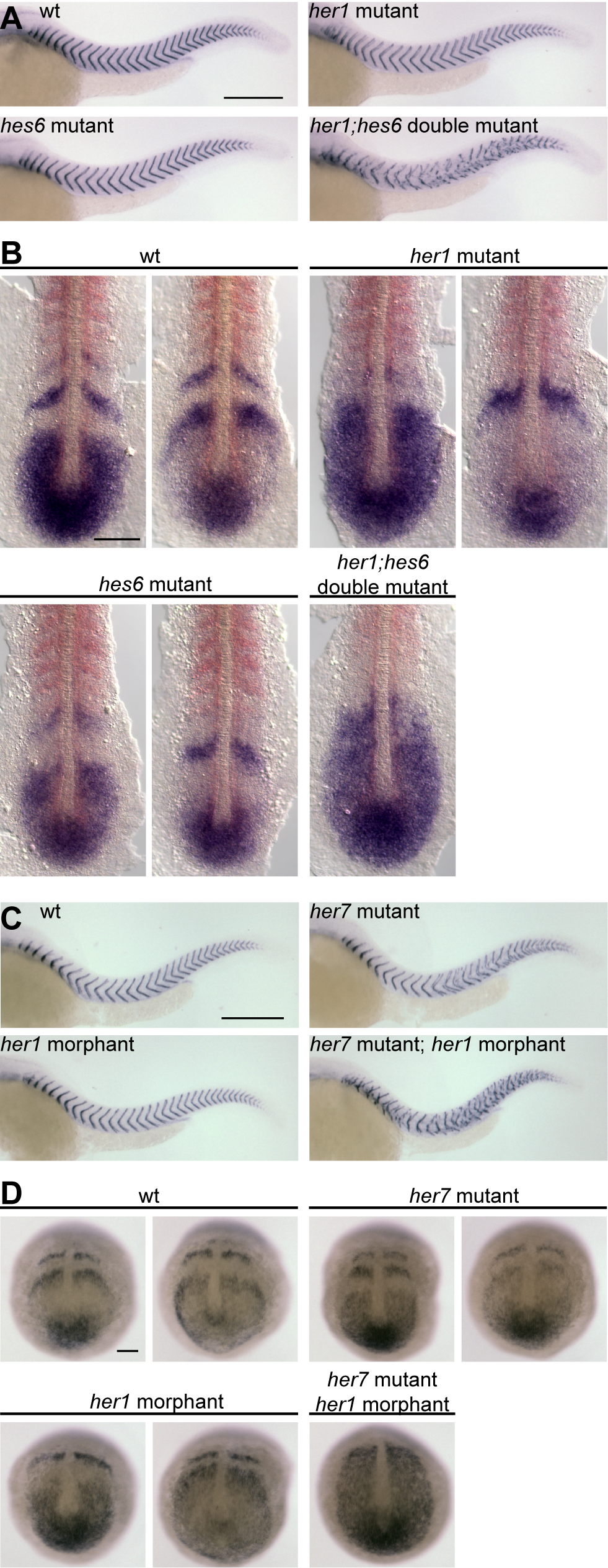

Supplement: Figure S6 — Combined loss of her1 and hes6 or her1 and her7 function fully disrupts segmentation and tissue-level oscillatory her7 expression. (A) Wildtype (wt), her1 mutant, hes6 mutant, and her1;hes6 double mutant embryos grown to 34 hpf and stained with the myotome boundary marker cb1045 to analyze segmentation. wt and the majority of her1 and hes6 single mutant embryos segment normally along the entire axis, whereas all her1;hes6 double mutant embryos display segmentation failure along the entire axis. Scale bar, 300 µm. (B) wt, her1 mutant, hes6 mutant, and her1;hes6 double mutant embryos at the 10-somite stained for her7 mRNA expression. Alternating wave patterns indicative for tissue-level oscillatory expression can be observed for wildtype, her1, and hes6 single mutant embryos (two representative examples shown for each genotype), but 26 out of 27 her1;hes6 double mutants display an equal level of her7 expression throughout the PSM. Scale bar, 100 µm. (C) wt and her7 mutant embryos were injected with her1-targeted MOs or left uninjected, grown until 34 hpf, and stained with the myotome boundary marker cb1045 to analyze segmentation phenotypes. All wildtype and the majority of her1 morphant embryos segment normally in the central trunk and tail, whereas her7 mutants display posterior segmentation defects. These defects are enhanced by injection of her1-targeted MOs into the mutant background, which leads to segmentation failure along the entire axis. Scale bar, 300 µm. (D) Uninjected and her1-MO-injected wt and her7 mutant embryos at bud-stage stained for her7 mRNA expression. Alternating wave patterns indicative for tissue-level oscillatory expression can be observed for uninjected and her1-MO-injected wt and uninjected her7 mutant embryos (two representative examples per condition shown), but her1 MO injection into her7 mutants leads to even her7 expression throughout the PSM (40 out of 40, one representative example shown). Scale bar, 100 µm. (TIF) [file pbio.1001364.s006.tif]

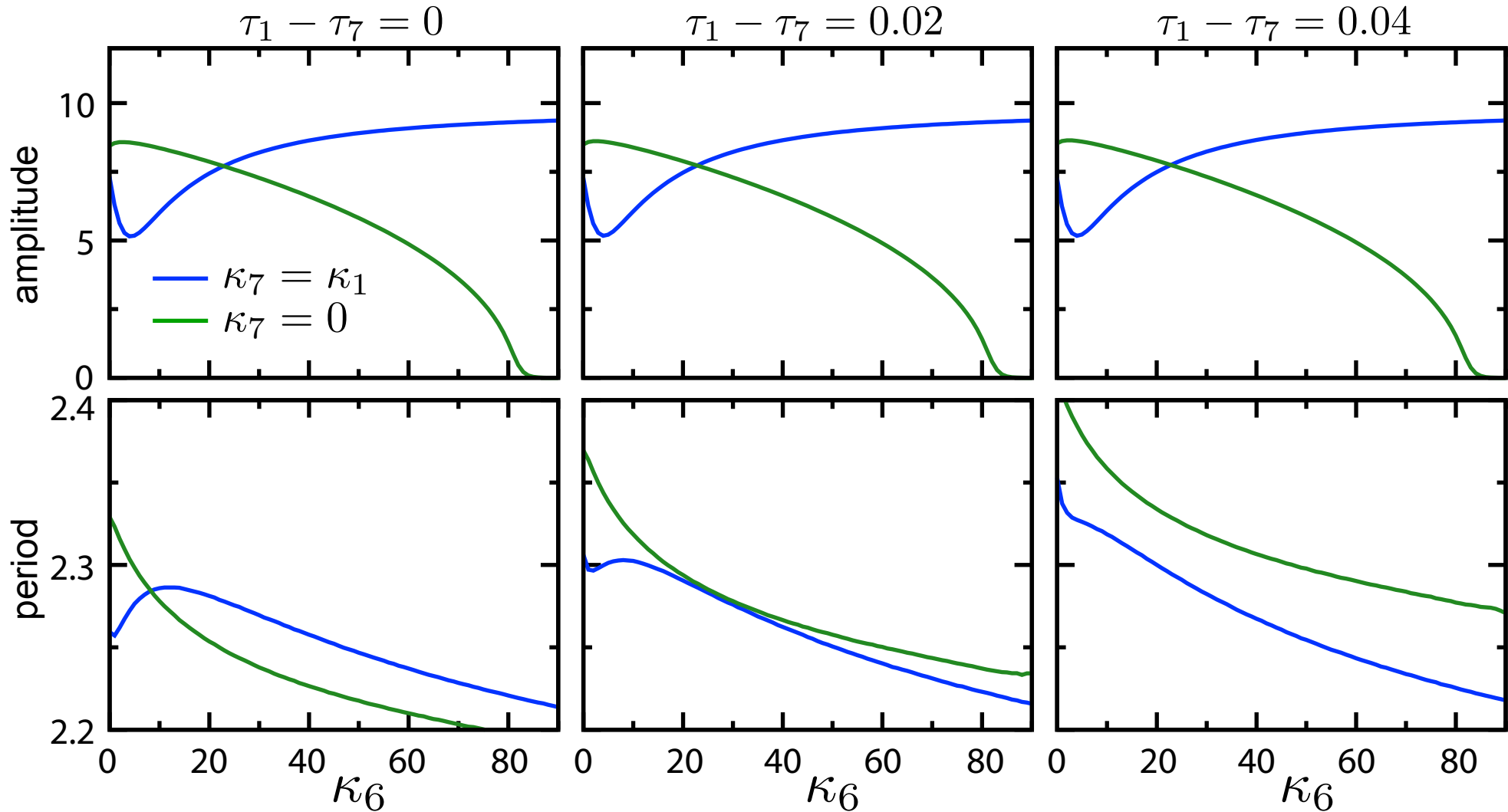

Supplement: Figure S7 — The difference between the production delays of Her1 and Her7 tunes the difference between wildtype and her7 loss-of-function periods. Amplitude (first row) and period (second row) of the oscillations of total Her1 protein concentration h 1, as a function of the dimensionless production rate of Hes6, κ 6, for three different values of the dimensionless production delay of Her1: τ 1 = 1∶00 (first column), τ 1 = 1∶02 (second column), and τ 1 = 1∶04 (third column). The amplitude is defined as the maximum minus the minimum of h 1 at steady state. All the other parameters as given in Table S3 for the blue line; same for the green line except κ 7 = 0. (PDF) [file pbio.1001364.s007.pdf]

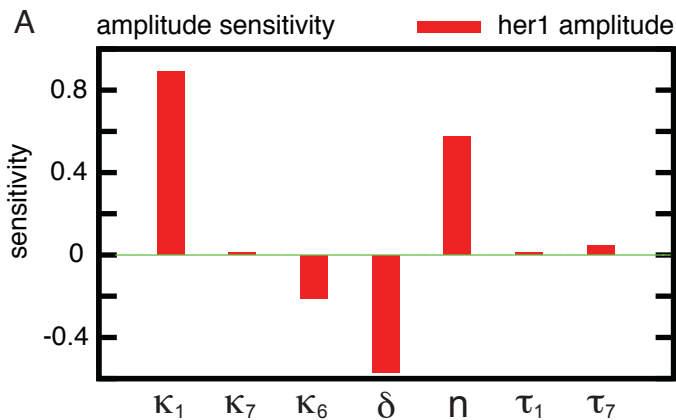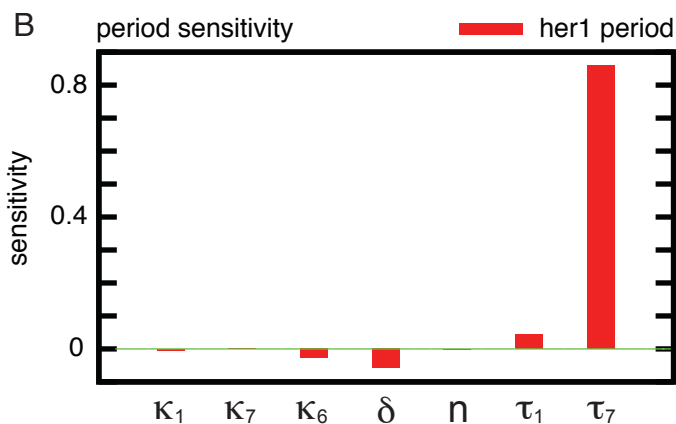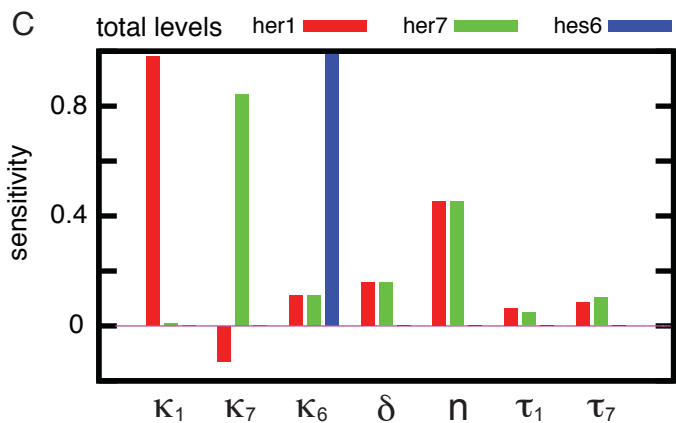

Supplement: Figure S8 — Sensitivity analysis shows that the model is robust to changes in parameters around the values chosen to describe the wildtype condition. See accompanying Text S1 for details. (PDF) [file pbio.1001364.s008.pdf]

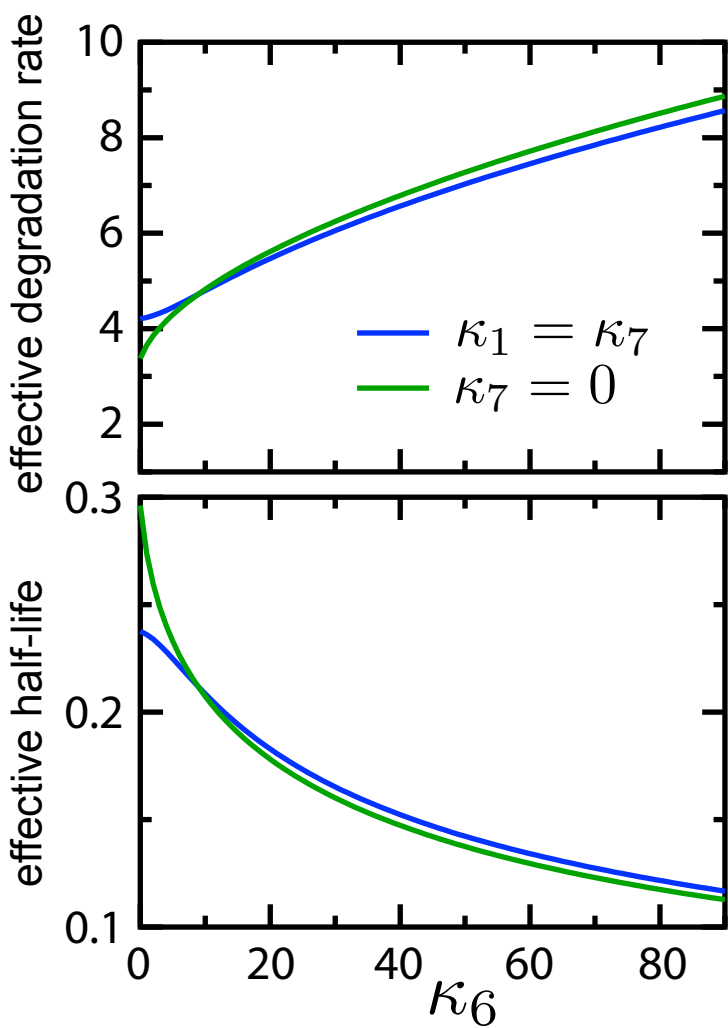

Supplement: Figure S9 — The dimensionless production rate of Hes6 can change the effective degradation rate of monomers. Average effective degradation rate (top) and average effective half-life (bottom) of Her1 monomer, as defined in Eq. (24). All the other parameters as given in Table S3 for the blue line, and same for the green line except κ 7 = 0. (PDF) [file pbio.1001364.s009.pdf]

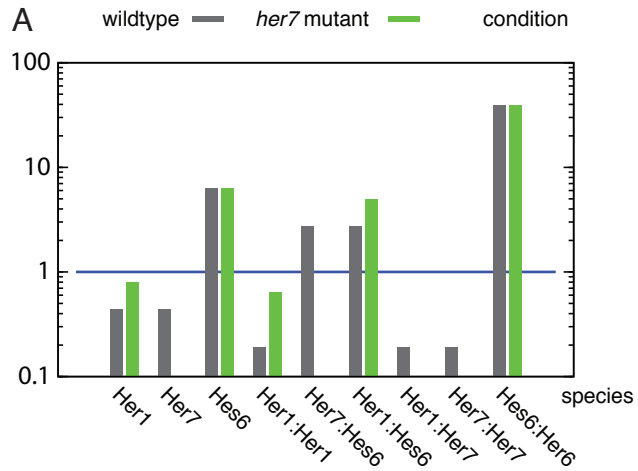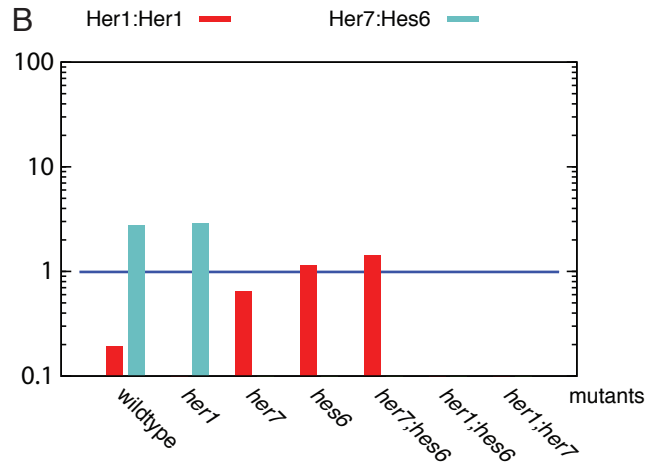

Supplement: Figure S10 — Average levels of Hes/Her monomers and dimers in wildtype and mutant conditions. (A) Comparison between the wildtype (grey bars) and the her7 mutant condition (green bars). Levels of all monomers and dimers are shown. (B) Levels of Her1 homodimer (red bars) and Her7:Hes6 heterodimer (cyan bars) in the different mutant conditions. The blue line shows the level at which negative feedback halves the production rate of Her1 and Her7. (PDF) [file pbio.1001364.s010.pdf]

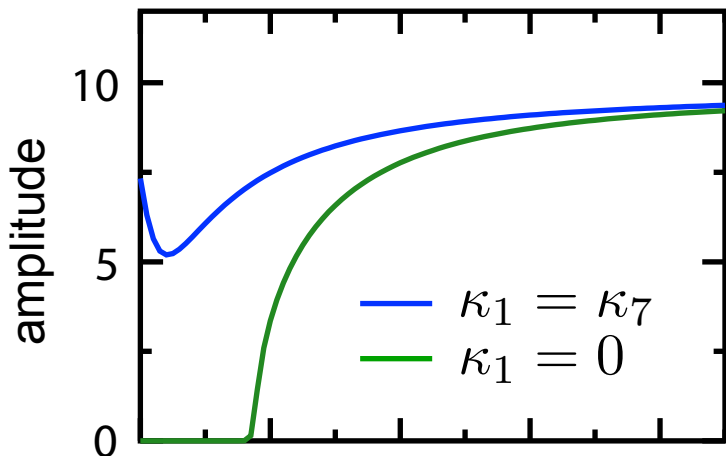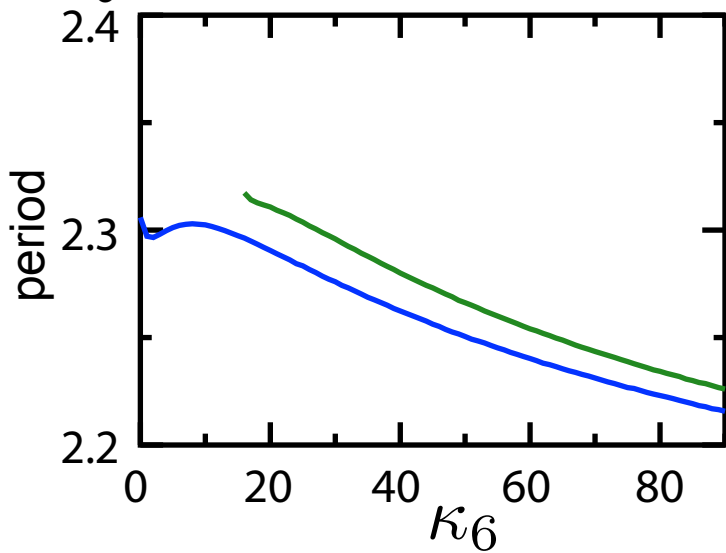

Supplement: Figure S11 — her1 loss of function has a similar period to wildtype. Amplitude (top) and period (bottom) of the oscillations of total Her7 protein concentration h 7, as a function of the dimensionless production rate of Hes6, κ 6. The amplitude is defined as the maximum minus the minimum of h 7 at steady state. All the other parameters as given in Table S3 for the blue line, and same for the green line except κ 1 = 0. (PDF) [file pbio.1001364.s011.pdf]

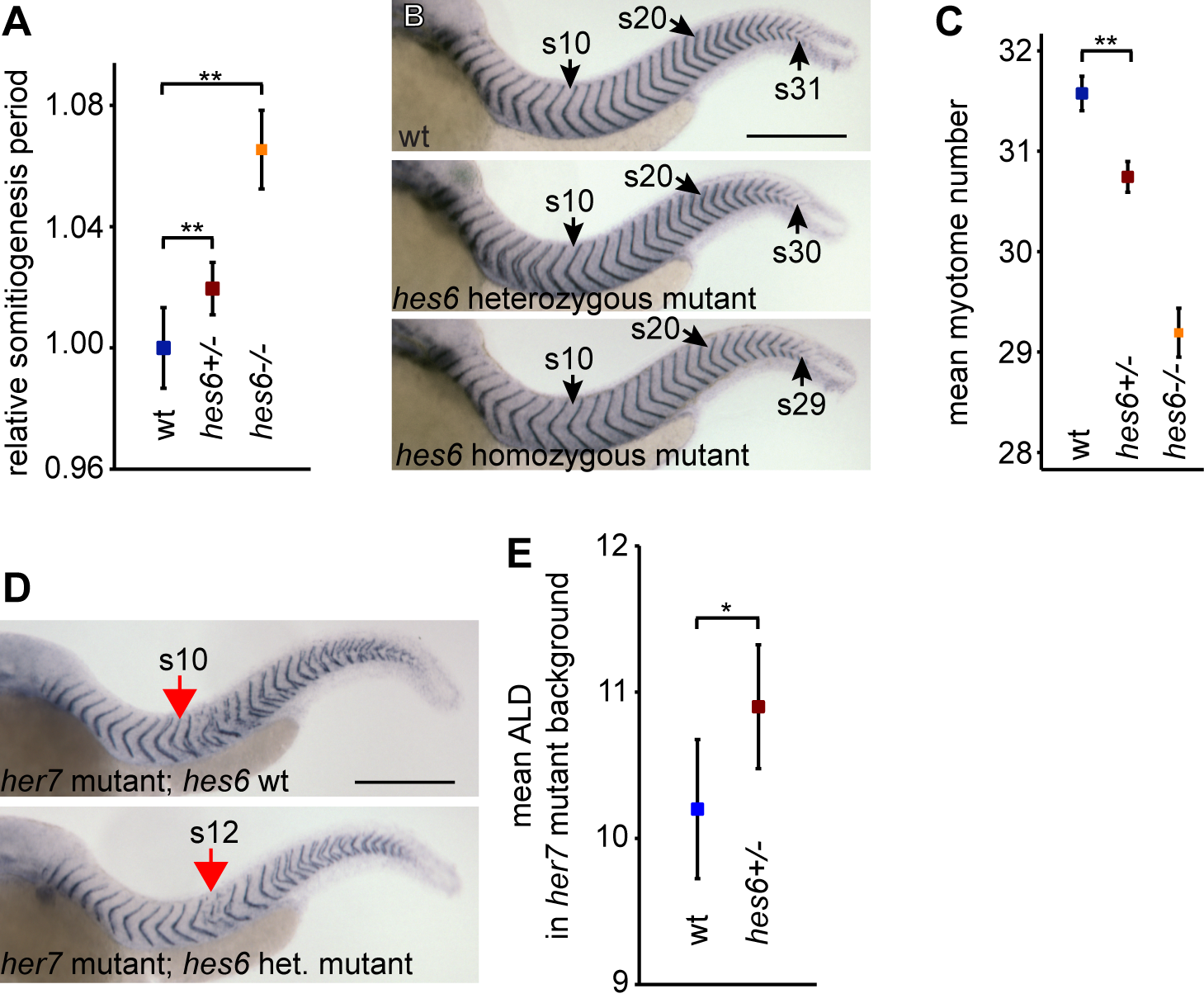

Supplement: Figure S12 — Quantitative effects of hes6 dosage on clock function. (A) Somitogenesis period measured by time-lapse imaging of embryos obtained from incrosses of heterozygous hes6 mutants. Period of wildtype (wt) embryos were normalized to one, and period of homozygous hes6 mutants is from [17], for comparison. Data pooled from three independent experiments, n≥19 for each genotype. Heterozygous hes6 mutants segment 2% slower than their wt siblings, while homozygous hes6 mutants segment 6% slower. (B) wt, heterozygous, and homozygous hes6 mutants at 48 hpf stained for cb1045 expression to count myotome number. The 10th, 20th, and last myotome is indicated for each genotype. (C) Quantification of myotome number in embryos stained as in (B) from incrosses of heterozygous hes6 mutants. Myotome number was scored by an observer blind to the embryos' genotype. Heterozgygous hes6 mutants have fewer segments than their wt siblings. Data pooled from two independent experiments, n≥26 per gentoype. (D) her7 homozygous mutants with a wt or heterozygous mutant hes6 locus at 34 hpf stained for cb1045 expression to determine anterior limit of segmentation defects (ALD, red arrow). (E) Quantification of ALD in embryos from an incross of her7 homozygous;hes6 heterozygous mutants. ALDs were scored by an observer blind to the embryos' genotype. The onset of segmentation defects in hes6 heterozygous her7 mutants is shifted toward the posterior compared to her7 mutants with two wildtype hes6 alleles. Data shown are from one representative experiment, n≥15 per genotype. ** and * indicate p≤0.01 and p≤0.05, respectively, as determined by two-tailed Mann-Whitney U-test. Error bars indicate 95% confidence interval. Scale bars 300 µm. (TIF) [file pbio.1001364.s012.tif]
