# Supplementary material for: Topology and Dynamics of the Zebrafish Segmentation Clock Core Circuit
Source: PLoS Biol. 2012 Jul 24;10(7):e1001364. doi: 10.1371/journal.pbio.1001364 (PMC3404119; doi:10.1371/journal.pbio.1001364)
Supplement: Table S2 — Parameters of the full model, Eqs. (1–9). (PDF) [file pbio.1001364.s014.pdf]

Table S2. Parameters of the full model, Eqs. (1-9).

Parameter	Description
k_μ	production rate (transcription + translation) of the monomer
$x_{\mu\nu}$	concentration of dimer that halves production rate
n_μ	phenomenological Hill coefficients describing effective cooperativity
c_μ	degradation rate of the monomer
$a_{\mu\nu}$	rate at which Herμ and Herν monomers form dimers
$b_{\mu\nu}$	rate at which the dimer Herμ:Herν breaks into monomers
$c_{\mu\nu}$	degradation rate of the dimer
τ_μ	production delay of the monomer
