# Supplementary material for: Topology and Dynamics of the Zebrafish Segmentation Clock Core Circuit
Source: PLoS Biol. 2012 Jul 24;10(7):e1001364. doi: 10.1371/journal.pbio.1001364 (PMC3404119; doi:10.1371/journal.pbio.1001364)
Supplement: Table S3 — Parameters of the minimal model in Eqs. (14–16). (PDF) [file pbio.1001364.s015.pdf]

Table S3. Parameters of the minimal model in Eqs. (14-16). The values used in our simulations are shown. For κ_6 we studied a range of values.

Parameter	Description	Value or range
κ_1	Her1 dimensionless production rate	10
κ_7	Her7 dimensionless production rate	10
κ_6	Hes6 dimensionless production rate	0 - 91
δ	dimensionless effective dimer mediated degradation rate	1
τ_1	dimensionless Her1 production delay	1.02
τ_7	dimensionless Her7 production delay	1.00
n	phenomenological Hill coefficients describing effective cooperativity	2
