# Supplementary material for: Topology and Dynamics of the Zebrafish Segmentation Clock Core Circuit
Source: PLoS Biol. 2012 Jul 24;10(7):e1001364. doi: 10.1371/journal.pbio.1001364 (PMC3404119; doi:10.1371/journal.pbio.1001364)
Supplement: Text S1 — Minimal asymmetric model of the gene regulatory network of the zebrafish segmentation clock. Includes Figures S7, S8, S9, S10, S11; Table S2: Parameters of the full model, Eqs. (1–9); and Table S3: Parameters of the minimal model, Eqs. (14–16). (PDF) [file pbio.1001364.s016.pdf]

Minimal asymmetric model of the gene regulatory network of the zebrafish segmentation clock

Christian Schröter[1,2,7], Saúl Ares[2,8,10] Luis G. Morelli[1,3,10] Alina Isakova[4] Korneel Hens[4] Daniele Soroldoni[1] Martin Gajewski[5,9] Frank Jülicher[2] Sebastian J. Maerkl[4,6] Bart Deplancke[4] Andrew C. Oates[1,*]
1 Max Planck Institute of Molecular Cell Biology and Genetics, Pfotenhauerstr. 108, D-01307 Dresden, Germany
2 Max Planck Institute for the Physics of Complex Systems, Nöthnitzer Str., 01187 Dresden, Germany
3 CONICET, Departamento de Física, Universidad de Buenos Aires, Buenos Aires, Argentina
4 École Polytechnique Fédérale de Lausanne, Institute of Bioengineering, Station 19, CH-1015 Lausanne, Switzerland
5 Institute for Genetics, University of Cologne, Zülpicher Str. 47, 50674 Cologne, Germany
6 École Polytechnique Fédérale de Lausanne, School of Engineering, Bldg. BM2111, Station 17, CH-1015 Lausanne, Switzerland
7 Current address: University of Cambridge, Department of Genetics, Downing Street, Cambridge CB2 3EH, UK
8 Current address: Logic of Genomic Systems Laboratory, Centro Nacional de Biotecnología - CSIC, Calle Darwin 3, 28049 Madrid, Spain
9 Current address: Hamilton Bonaduz AG, Via Crusch 8, 7402 Bonaduz, Switzerland
10 These authors contributed equally
∗ E-mail: Corresponding oates@mpi-cbg.de

Abstract

We propose a dynamical model for the core circuit of the single cell genetic oscillator of the zebrafish segmentation clock, describing the expression levels of Her1, Her7 and Hes6 proteins and the dimers they form. The regulatory interactions in the model are constrained by the biochemical data discussed in the Main Text. Starting from a full model that describes dimerization explicitly, and assuming that dimerization is much faster than other reactions, we obtain a simplified model that incorporates the dimer dynamics as an effective degradation term of monomers. We use numerical solutions of this simplified model to show that the biochemical interactions discussed in the Main Text are sufficient to explain the phenotypes observed in the single and double mutants.

1 Full model explicitly including dimerization

We use the data we obtained from protein-protein and protein-DNA binding assays for the *her1*, *her7* and *hes6* genes (from here on, the her genes), to develop a mathematical model of the gene network that drives single cell oscillations in the segmentation clock. The key observations that constrain the architecture of the model are:

- Dimer formation is promiscuous: all Her proteins make dimers with all other Her proteins, Figure 1 Main Text.
- The Her1 homodimer and the Her7:Hes6 heterodimer are the strongest DNA binding dimers, targeting the regulatory regions of *her1* and *her7*, Figures 1-3 Main Text. Other dimers have a much weaker DNA binding activity or no detectable binding.

- Both *her1* and *her7* show cyclic gene expression patterns and are thought to be transcriptionally regulated, but *hes6* mRNA expression does not oscillate, see Figure 8 Main Text.

Following this evidence, we propose a model where all the possible dimers form, but only the Her1 homodimer and the Her7:Hes6 heterodimer perform regulatory functions as repressors of *her1* and *her7* expression, see Figure 7A Main Text. Since *her1* and *her7* promoters have similar target sites, Figures 2 and 3 Main Text, we assume that they are both regulated in the same way by binding of the Her1 homodimer and the Her7:Hes6 heterodimer. *hes6* synthesis rate is constant, Figure 8 Main Text.

We use delay differential equations to describe the dynamics of molecular species concentrations. The model consists of nine equations, three for the monomers of the Her proteins in the model, and six more to account for all its possible dimers:

$$\frac{dH_1(t)}{dt} = \frac{k_1}{1+\left(\frac{H_{11}(t-\tau_1)}{x_{11}}+\frac{H_{76}(t-\tau_1)}{x_{76}}\right)^{n_1}} - c_1 H_1(t) \tag{1}$$
$$- 2a_{11}H_1(t)^2 + 2b_{11}H_{11}(t) - a_{16}H_1(t)H_6(t) + b_{16}H_{16}(t) - a_{17}H_1(t)H_7(t) + b_{17}H_{17}(t)$$

$$\frac{dH_6(t)}{dt} = k_6 - c_6 H_6(t) \tag{2}$$
$$- 2a_{66}H_6(t)^2 + 2b_{66}H_{66}(t) - a_{16}H_1(t)H_6(t) + b_{16}H_{16}(t) - a_{76}H_6(t)H_7(t) + b_{76}H_{76}(t)$$

$$\frac{dH_7(t)}{dt} = \frac{k_7}{1+\left(\frac{H_{11}(t-\tau_7)}{x_{11}}+\frac{H_{76}(t-\tau_7)}{x_{76}}\right)^{n_7}} - c_7 H_7(t) \tag{3}$$
$$- 2a_{77}H_7(t)^2 + 2b_{77}H_{77}(t) - a_{76}H_7(t)H_6(t) + b_{76}H_{76}(t) - a_{17}H_1(t)H_7(t) + b_{17}H_{17}(t)$$

$$\frac{dH_{11}(t)}{dt} = a_{11}H_1(t)^2 - b_{11}H_{11}(t) - c_{11}H_{11}(t) \tag{4}$$

$$\frac{dH_{16}(t)}{dt} = a_{16}H_1(t)H_6(t) - b_{16}H_{16}(t) - c_{16}H_{16}(t) \tag{5}$$

$$\frac{dH_{17}(t)}{dt} = a_{17}H_1(t)H_7(t) - b_{17}H_{17}(t) - c_{17}H_{17}(t) \tag{6}$$

$$\frac{dH_{66}(t)}{dt} = a_{66}H_6(t)^2 - b_{66}H_{66}(t) - c_{66}H_{66}(t) \tag{7}$$

$$\frac{dH_{76}(t)}{dt} = a_{76}H_7(t)H_6(t) - b_{76}H_{76}(t) - c_{76}H_{76}(t) \tag{8}$$

$$\frac{dH_{77}(t)}{dt} = a_{77}H_7(t)^2 - b_{77}H_{77}(t) - c_{77}H_{77}(t) \tag{9}$$

where $H_\mu(t)$ denotes the concentration of the Herμ monomer, and $H_{\mu\nu}(t)$ is the concentration of the Herμ:Herν dimer[1]:

$$H_\mu(t) \equiv [\text{Her}\mu]\,, \tag{10}$$

$$H_{\mu\nu}(t) \equiv [\text{Her}\mu\text{:Her}\nu]\,. \tag{11}$$

The indexes μ and ν take on values among 1, 6, and 7, where Hes6 is denoted as Her6 for simplicity. Parameters in Eqs. (1-9) are listed and described in Table S2. To simplify the model, we write equations only for protein concentrations, and do not consider mRNA expression or intercellular compartmentalization explicitly [1,2]. It has been shown that the qualitative behavior of amplitude and period in models of genetic oscillators is rather insensitive to these details [3]. The time necessary for transcription and translation, as well as intracellular transport, is taken into account phenomenologically by introducing

[1] For instance, $H_1(t)$ is the concentration of Her1, H_{11} is the concentration of the Her1 homodimer, H_{16} the concentration of the Her1:Hes6 heterodimer, and so on.

time delays in the regulatory terms of the equations for the monomers Her1 and Her7. For simplicity, we assume that degradation of products is linear.

Biochemical regulatory networks require a delayed negative feedback together with some non-linearity, in order to support sustained oscillations [4]. Using MITOMI and Y1H assays, we experimentally identified multiple Hes/Her binding sites in cyclic gene promoters, Figures 2C and 3D Main Text. The effects of multiple binding sites with different affinities in each promoter can be described by a Hill function with effective cooperativity [5, 6]. Motivated by this observation, in our model negative regulation through Her1 homodimers and Her7:Hes6 heterodimers is represented by a Hill function with effective cooperativity n_μ. In our model, this non-linearity of the feedback with an explicit time delay is sufficient to drive oscillations.

Since the binding energy landscapes of the Her1 homodimer and the Her7:Hes6 heterodimer are similar (Figure 2 Main Text), we assume that the promoters do not distinguish among them. However, there is currently no data constraining the repressive activity of the dimers, and therefore in this full model we allow for different repressive strengths represented by different values of x_{11} and x_{76}. We also assume that the production rate of Hes6 is constant, justified by the observation that its mRNA expression is constant in time at any given point within the PSM [7, 8], Figure 8 Main Text.

Since we focus here on the single cell oscillator, we do not include Delta/Notch signaling in the model. Furthermore, we do not describe spatial extension and the patterns of oscillating gene expression across the PSM. The model therefore describes an uncoupled cell in the posterior pre-somitic mesoderm or tailbud. It will be interesting to explore the effects of intercellular coupling and spatial regulation of oscillators. To focus on the the core characteristics of the model, in this work we only consider a deterministic implementation of the model.

2 Derivation of the minimal model

In order to simplify the analysis, here we reduce the full model into a simpler minimal model. We first take the parameters corresponding to different molecular species in the model to be equal. In this way we reduce the differences in parametrization between species, and any asymmetry appearing in the behavior of different species will arise as an effect of the network's topology. Then we convert the equations into non-dimensional form. This has two advantages: first, the number of parameters is further reduced and the results can be expressed in terms of scaled variables; second, a consistent equilibrium approximation can be used to simplify the dynamics of dimerization [9].

In the following, we take $a_{\mu\nu} \equiv a$ and $b_{\mu\nu} \equiv b$ for all species in the model. This means that the rates of dimerization and its inverse reaction are independent of the species. Similarly, we take $c_{\mu\nu} \equiv c$ and $c_\mu \equiv c$, i.e. degradation rates are the same for all monomers and dimers. Finally, we set $x_{11} = x_{76} = x$ and $n_1 = n_7 = n$. Together, these equalities mean that *her1* and *her7* are repressed with the same strength, both by Her1 homodimers and Her7:Hes6 heterodimers. While some of these simplifications may not reflect the physiological reality in the fish, here we use them to test whether the asymmetry in the network is enough to explain the observed phenotypes.

After these simplifications, the only concentration scale in the model is x, the concentration of dimers that halves the transcription rate. We use this concentration scale and the timescale given by the inverse of the degradation rate c to non-dimensionalize the model [10], introducing a rescaled time $s \equiv ct$, the parameters $\alpha \equiv ax/c$, $\beta \equiv b/c$, $\delta \equiv (\alpha/(1+\beta))^{1/2}$, $\kappa_\mu \equiv k_\mu \delta/cx$ and $\tau_\mu \equiv c\mathrm{T}_\mu$, and dimensionless concentrations $h_\mu \equiv \delta H_\mu/x$ and $h_{\mu\nu} \equiv \delta H_{\mu\nu}/x$. The non-dimensional equation for dimer dynamics is:

$$\frac{1}{\alpha}\frac{dh_{\mu\nu}(s)}{ds} = \frac{1}{\delta}h_\mu(s)h_\nu(s) - \frac{1}{\delta^2}h_{\mu\nu}(s)\,. \tag{12}$$

Assuming that dimerization reactions (binding and unbinding of monomers) are much faster than degradation [11], we can adiabatically eliminate the dimer concentrations. Formally, we take $b \gg c$ and $ax \gg c$,

so $\alpha \gg 1$ while δ is of order unity, $\delta \sim \mathcal{O}(1)$. This separation of time scales implies that

$$h_{\mu\nu}(s) = \delta h_\mu(s) h_\nu(s)\,. \tag{13}$$

Note however that the time derivative of the rescaled dimer concentration need not vanish in Eq. (12). Another way to interpret this is to note that $dh_{\mu\nu}/ds$ is large everywhere, except very close to the surface defined by Eq. (13), also known as the slow manifold of the system. Therefore, dimer concentrations very quickly relax to the value given by Eq. (13), and are determined by the monomer concentrations, as these change on a slower time scale.

3 Minimal model with effective degradation

With the simplifications outlined in the previous section, the resulting minimal model is:

$$\begin{aligned} \frac{dh_1(s)}{ds} =& \frac{\kappa_1}{1+\left(h_1(s-\tau_1)^2+h_7(s-\tau_1)h_6(s-\tau_1)\right)^n} - h_1(s) \\ & -\delta\; h_1(s)\left(2h_1(s)+h_6(s)+h_7(s)\right) \end{aligned} \tag{14}$$

$$\begin{aligned} \frac{dh_6(s)}{ds} =& \kappa_6 - \;h_6(s) \\ & -\delta\; h_6(s)\left(h_1(s)+2h_6(s)+h_7(s)\right) \end{aligned} \tag{15}$$

$$\begin{aligned} \frac{dh_7(s)}{ds} =& \frac{\kappa_7}{1+\left(h_1(s-\tau_7)^2+h_7(s-\tau_7)h_6(s-\tau_7)\right)^n} - \;h_7(s) \\ & -\delta\; h_7(s)\left(h_1(s)+h_6(s)+2h_7(s)\right)\,. \end{aligned} \tag{16}$$

Parameters are listed and described in Table S3. Dimers mediate the degradation of monomers, as represented by effective degradation terms in Eqs. (14-16), and their dynamics is given by Eq. (13). These non-linear degradation terms cross-regulate the dynamics of the different monomers. If one of the concentrations oscillates, the effective degradation rate of the others will also display oscillations, introducing a post-translational level of regulation.

The minimal model Eqs. (14-16) is a strong simplification of the full model Eqs. (1-9). From 9 equations and 30 parameters, we now have 3 equations and only 6 parameters. Of these 6 parameters, two are the time delays and one is the effective Hill exponent, leaving only three independent dimensionless rates. The minimal model is still asymmetric with respect to Her1 and Her7: Her1 binds as a homodimer, while Her7 forms a heterodimer with Hes6. Except for the time delays, this asymmetry is purely topological, and it persists even if we make all the rates equal in Eqs. (14-16).

We will explore how far one can get with this setting, but we should keep in mind that the full model Eqs. (1-9) allows for a much larger variety of asymmetries between molecular species, appearing not only from the asymmetric topology of the network but also from the differences in the kinetics between different species and their biochemical activities.

4 Numerical solutions of the minimal model

We obtained numerical solutions of our model using MATLAB function dde23. As initial condition, we set the concentrations of all species to zero. In order to track the total amount $\bar{h}_\mu$ of each of the Her proteins in the system, we need to count the amount present in dimers:

$$\bar{h}_1(s) = h_1(s) + 2h_{11}(s) + h_{16}(s) + h_{17}(s) \tag{17}$$

$$\bar{h}_6(s) = h_6(s) + 2h_{66}(s) + h_{16}(s) + h_{76}(s) \tag{18}$$

$$\bar{h}_7(s) = h_7(s) + 2h_{77}(s) + h_{76}(s) + h_{17}(s) \tag{19}$$

where the dimer concentrations are given by Eq. (13). These variables $\bar{h}_\mu$ allow to quantitate the amplitude of the oscillations of protein concentrations in Figure S7, irrespective of the individual protein molecules being monomers or part of dimers. The qualitative behavior of the oscillations is well represented by the cases displayed in Figure S7 for an effective Hill exponent $n = 2$. In this figure we see that in general the period of the oscillations decreases with an increasing value of the dimensionless production rate of Hes6, κ_6. This is consistent with the period of the Hes6 mutant being higher than the wildtype period, [8] and Figure 6 Main Text.

5 Sensitivity analysis and robustness

In the Main Text, we have chosen parameters that describe the wildtype condition and the behavior of mutants, Figure 7 Main Text. We will discuss in coming sections how these parameters were chosen. An important question is how the observables of the system, like the period and amplitude of oscillations, depend on parameters. In particular, it is important to know if such observables, that we use to characterize the behavior in different conditions, are sensitive to small changes in parameter values. To address this question we perform a sensitivity analysis, as introduced by Savageau [12,13]. We define the sensitivity $S_{x\alpha}$ of the observable X with respect to changes in parameter α as

$$S_{x\alpha} = \frac{\partial \ln X}{\partial \ln \alpha} \,. \tag{20}$$

This quantity is evaluated at some point in parameter space, that is, for some fixed values of all parameters in the model. Here we compute the sensitivity at the point in parameter space that we have chosen to describe wildtype behavior, as summarized in Table S3, with $\kappa_6 = 90$.

The logarithmic derivative can be re-written as

$$S_{x\alpha} = \frac{dX/X}{d\alpha/\alpha} \,. \tag{21}$$

Therefore, to compute the sensitivity, we approximate the logarithmic derivative by the ratio of relative changes

$$S_{x\alpha} = \frac{\Delta X/X}{\Delta\alpha/\alpha} \,. \tag{22}$$

We determine the sensitivity at α_0 by evaluating the observable at three points: the focal point α_0, and $\alpha = \alpha_0 \pm 0.05\,\alpha_0$. The sensitivity thus defined is the percentage change in the variable given a percentage change in the parameter.

We focus on five key observables, the amplitude of Her1 oscillations, the period of oscillations, and the average total levels of the three species, Her1, Her7 and Hes6. The results are shown in Figure S8 and show that the observables are very robust to changes in parameters. The amplitude of oscillations is affected by Her1 production rate κ_1, the effective degradation rate δ, and the effective cooperatively n, Figure S8A. For this parameter set, the period of oscillations is most sensitive to changes in τ_7, Figure S8B. The delays in the negative feedback loops are a main contribution to the period in a delayed feedback oscillator [4, 14]. Finally, the average levels of different species are affected mainly by the respective production rates, and only very weakly by other parameters, Figure S8C. Apart from these observations, the main conclusion of Figure S8 is that the behavior of the system is robust, showing a relatively weak sensitivity to all parameters.

6 Determination of the dimensionless production rate of Hes6

To choose the value of the dimensionless production rate of Hes6 κ_6 corresponding to the wildtype condition, we start with $\kappa_6 = 0$ describing the *hes6* mutant condition and increase the value of κ_6 until

the difference in period with respect to $\kappa_6 = 0$ reflects the experimentally observed difference between wildtype and *hes6* mutant, Figure 6 Main Text. At $\kappa_6 = 90$, the *her7* loss of function situation ($\kappa_7 = 0$) results in a dramatic drop of amplitude and loss of sustained oscillations, Figure S7 green curves. This gives an explanation for the *her7* mutant condition, which displays a decay of oscillatory activity. We analyze the origin of these damped oscillations in our simulated her7 mutant in Section 9 below. As κ_6 decreases in a *her7* mutant background (green curves), sustained oscillations reappear and the amplitude of oscillations increases, Figure S7 upper panels. The striking rescue of the *her7* decay phenotype due to additional mutation of *hes6* is thus explained in the model by the full recovery of oscillations for $\kappa_6 = 0$ in a $\kappa_7 = 0$ situation.

7 Hes6 changes the period by controlling effective half-lives

The period is increased in a *hes6* mutant condition $\kappa_6 = 0$. More generally, the dimensionless production rate of Hes6 can smoothy control the period of the oscillations, Figure S7. This grip of *hes6* on the period can be understood in terms of the effective half-life of the monomers, defined as the sum of loss terms in Eqs. (14-16), divided by the corresponding monomer concentration. For example, for Her1:

$$d_1(s) = 1 + \delta\left(2h_1(s) + h_6(s) + h_7(s)\right). \tag{23}$$

The effective degradation rate $d_\mu(s)$ is a function of dimensionless time s because the monomer concentrations $h_1(s)$, $h_7(s)$, and $h_6(s)$, can oscillate. We therefore introduce its time average $\bar{d}_\mu$,

$$\bar{d}_\mu = \frac{1}{S}\int_S d_\mu(s)\,ds \tag{24}$$

where the average is performed over a complete cycle of the oscillator, and S is the dimensionless period. In the absence of dimerization ($\delta = 0$) the effective decay rate is simply the decay rate of the monomer, which is one in the dimensionless form of the minimal model.

We obtained numerical solutions of the minimal model and computed the average effective degradation rate for Her1 monomer, Figure S9 top panel. The effective degradation rate smoothly increases with κ_6. As Hes6 monomer becomes more abundant, it binds Her1 monomers and increases Her1 degradation rate. The effective half-life, defined here as the inverse of the effective degradation rate, decreases correspondingly, Figure S9 bottom panel. The period of a biochemical oscillator is mainly determined by the feedback delay and the half-life of the repressors [4]. Most of the change in period in the *hes6* mutant appears to come from this change in effective half-life.

8 Determination of the time delays in Her1 and Her7 production

To choose the values of the production delays in our model, we looked for values of τ_1 and τ_7 for which the periods of a wildtype-like situation ($\kappa_1 = \kappa_7$) and a *her7* loss of function situation ($\kappa_7 = 0$) coincide over a wide range of κ_6, the dimensionless production rate of Hes6. For the parameter values listed in Table S3, this constraint is satisfied when $\tau_1 = 1.02$ and $\tau_7 = 1.00$ (Figure S7, second column). For smaller differences between τ_1 and τ_7, the period in the absence of Her7 activity is generally smaller (Figure S7, first column), and for larger differences the period in the absence of Her7 is larger (Figure S7, third column). Although chosen to match the experimental data, qualitatively this difference in the delays is justified molecularly because *her7* is a shorter gene, encoding a shorter protein, and has less introns than *her1*. Since the delays arise in part from splicing and sequential polymerization reactions in transcription and translation, this results in shorter production delays for Her7 than Her1.

Quantitatively, the relatively small difference appears consistent with independent experimental data, which has established that transcription is fast (4 kb/min), intron splicing is co-transcriptional, splicing times are short (5 min) and independent of length [15]. However, note that our choice of time delays is not an unavoidable property of the full model, since a similar effect may be achieved by introducing small differences between Her1 and Her7 in parameters other than the time delays.

9 Hes6 titrates the level of Her1 homodimer

The amplitude damping in a *her7* mutant condition can be explained by the molecular titration of Her1 homodimers by Hes6. Molecular titration occurs when an active protein is sequestered into an inactive complex [16]. Here, Hes6 sequesters Her1 into Her1:Hes6 heterodimers with weak DNA-binding activity, obstructing the formation of strong DNA-binding Her1 homodimers, Figure 7A Main Text. This mechanism can be exposed by observation of the levels of dimers in different situations. Therefore, we introduce the average levels of monomers and dimers, defined by

$$\hat{h}_\mu = \frac{1}{S}\int_S h_\mu(s)\,ds \tag{25}$$

and

$$\hat{h}_{\mu\nu} = \frac{1}{S}\int_S h_{\mu\nu}(s)\,ds\,, \tag{26}$$

where a time average is taken over a complete cycle of the oscillator, of dimensionless period S. In the wildtype condition, grey bars in Figure S10A, Her1:Her1 levels are low compared to Her7:Hes6 levels. Upon loss of *her7*, green bars in Figure S10A, Her1 monomer levels go up due to loss of repression from the Her7:Hes6 feedback loop, Figure 7A Main Text. However, in this situation Hes6 sequesters most Her1 monomers into Her1:Hes6 heterodimers with weak DNA-binding activity, and the resulting Her1 homodimer level is not enough to sustain oscillations.

The levels of the two strong DNA-binding species, Her1 homodimers and Her7:Hes6 heterodimers, are displayed in Figure S10B for all mutant conditions. In the *hes6* mutant and the *her7;hes6* double mutant, the levels of Her1 homodimer are higher than the the level at which negative feedback halves the production rate of Her1 and Her7 (blue line), as compared to the *her7* mutant condition where Her1 homodimer level is below the half-repression level. This shows that when no regulatory interactions are involved, the presence of Hes6 acts to reduce the Her1 homodimer level in the system. Thus, Hes6 levels act as a dial and can switch between the Her1 homodimer loop and the Her7:Hes6 heterodimer loop in the model.

10 Her1 loss of function

In the main text we focus of the *her7* loss of function situation and its relation to *hes6* loss of function, Figure 7 Main Text. Here we expand the analysis and consider the *her1* loss of function situation in our model ($\kappa_1 = 0$). In Figure S11 (top) we see that for low values of κ_6, there are no oscillations in the $\kappa_1 = 0$ case, as was expected due to the lack of repression in the absence of Her1 and low levels of Her7:Hes6, Figure S6. However, for all the range where oscillations are possible, the period of the *her1* loss of function case is similar to the wildtype case, Figure S11 (bottom). We have repeated this simulation for different values of the production delay of Her1, τ_1, and found that the behavior displayed in Figure S11 depends very weakly on the difference between the production delays of Her1 and Her7. This means that the similarity between wildtype and *her1* loss of function periods is a general property of the model, in agreement with our experimental observations, Figure 6 Main Text.

References

1. Monk NAM (2003) Oscillatory expression of Hes1, p53, and NF-κB driven by transcriptional time delays. Curr Biol 13: 1409-1413.

2. MacDonald N (1989) Biological delay systems. Cambridge University Press.

3. Momiji H, Monk NAM (2008) Dissecting the dynamics of the Hes1 genetic oscillator. J Theor Biol 254: 784-798.

4. Novák B, Tyson JJ (2008) Design principles of biochemical oscillators. Nat Rev Mol Cell Biol 9: 981-991.

5. Rubinow SI, Segel LA (1991) Positive and negative cooperativity. In: Segel LA, editor, Biological Kineticss, Cambridge University Press. pp. 29-44.

6. Zeiser S, Liebscher HV, Tiedemann H, Rubio-Aliaga I, Przemeck GKH, et al. (2006) Number of active transcription factor binding sites is essential for the Hes7 oscillator. Theor Biol Med Model 3: 11.

7. Kawamura A, Koshida S, Hijikata H, Sakaguchi T, Kondoh H, et al. (2005) Zebrafish hairy/enhancer of split protein links FGF signaling to cyclic gene expression in the periodic segmentation of somites. Genes Dev 19: 1156-1161.

8. Schröter C, Oates AC (2010) Segment number and axial identity in a segmentation clock period mutant. Curr Biol 20: 1254-1258.

9. Keener J, Sneyd J (2009) Mathematical Physiology I: Cellular Physiology. Springer, 2nd. edition.

10. Strogatz SH (1994) Nonlinear Dynamics and Chaos. Reading, MA: Addison-Wesley.

11. Spinner DS, Liu S, Wang SW, Schmidt J (2002) Interaction of the myogenic determination factor myogenin with E12 and a DNA target: mechanism and kinetics. J Mol Biol 317: 431-445.

12. Savageau MA (1971) Parameter sensitivity as a criterion for evaluating and comparing performance of biochemical systems. Nature 229: 542-544.

13. Savageau MA (1971) Concepts relating behavior of biochemical systems to their underlying molecular properties. Archives Of Biochemistry And Biophysics 145: 612-621.

14. Lewis J (2003) Autoinhibition with transcriptional delay: a simple mechanism for the zebrafish somitogenesis oscillator. Curr Biol 13: 1398-1408.

15. Singh J, Padgett RA (2009) Rates of in situ transcription and splicing in large human genes. Nat Struct Mol Biol 16: 1128-1133.

16. Buchler NE, Louis M (2008) Molecular titration and ultrasensitivity in regulatory networks. J Mol Biol 384: 1106-1119.
